# Decoding AI Competence: Benchmarking Large Language Models (LLMs) in Ovarian Cancer Diagnosis and Treatment—A Systematic Evaluation of Generative AI Accuracy and Completeness

**DOI:** 10.3390/diagnostics16040616

**Published:** 2026-02-20

**Authors:** Haojie Cai, Chao Wang, Yue Zhang, Hui Ding, Wei Hong, Yaqian Zhao, Shanshan Cheng, Yu Wang

**Affiliations:** Department of Gynecology, Shanghai Key Laboratory of Maternal Fetal Medicine, Shanghai Institute of Maternal-Fetal Medicine and Gynecologic Oncology, Shanghai First Maternity and Infant Hospital, School of Medicine, Tongji University, Shanghai 200092, China; 2411166@tongji.edu.cn (H.C.); 13761183836@163.com (C.W.); yzhang2013@126.com (Y.Z.); dinghui_doctor@163.com (H.D.); hw_rock@sina.com (W.H.); yxszhaoyaqian@163.com (Y.Z.)

**Keywords:** DeepSeek, Doubao, ovarian cancer, large language models, artificial intelligence

## Abstract

**Objective**: To evaluate the practical value of DeepSeek-R1 and Doubao-1.5-pro in the context of ovarian cancer management by examining their diagnostic and treatment-related competencies. **Methods**: 20 key ovarian cancer diagnosis and treatment issues were identified, divided into 4 domains with 5 questions each. Two large language models answered these questions, and 5 gynecologic oncology chief physicians evaluated the answers on a 1–10 scale for completeness and accuracy. For each score and the mean score for each question, if it surpassed 7, it is evaluated as “Excellent.” The Kruskal–Wallis test compared scores within each LLM across 4 categories, and the Mann–Whitney-Wilcoxon test compared scores between the two LLMs in each category. **Results**: 200 scores were collected (100 per model). DeepSeek-R1 got 98 “Excellent” ratings, while Doubao-1.5-pro got 41. All 20 DeepSeek-R1 responses had “Excellent” average scores, compared to 9 for Doubao-1.5-pro. DeepSeek-R1 had less variability. Tests revealed significant differences between the models and showed DeepSeek-R1 outperformed Doubao-1.5-pro, and charts showed Doubao-1.5-pro scored lower in all aspects, especially “Medical”. **Conclusions**: DeepSeek-R1 shows potential in ovarian cancer diagnosis and treatment but has limitations like inaccuracies and overly technical responses due to outdated data and lack of humanistic elements. LLMs like DeepSeek-R1 are useful for medical education and assistive diagnosis, but they require ongoing updates and refinement for broader clinical use. Selecting the appropriate LLM for medical tasks and improving their clarity and accuracy is crucial for their future effectiveness.

## 1. Introduction

Ovarian cancer, one of the most aggressive gynecologic malignancies, remains a significant global health concern. In China, it has been the second leading cause of death among gynecologic cancers since approximately 2005 [1]. Globally, a substantial number of women succumb to ovarian cancer [2]. As such, the development of rational, timely, and effective diagnostic and therapeutic strategies for ovarian cancer has long been a priority for the medical community.

In recent years, rapid technological advancements have made artificial intelligence (AI) an increasingly indispensable tool across various sectors, particularly in healthcare [3]. Among the various AI domains, large language models (LLMs) have shown remarkable promise in medical applications and have already attracted a considerable amount of in-depth research [4,5,6,7,8,9,10]. For instance, Med-PaLM 2, an advanced LLM, has demonstrated diagnostic and treatment capabilities comparable to those of physicians, highlighting its significant potential in the medical field [11]. Scientists have also explored the broad potential applications of LLMs in diagnosing cardiovascular diseases, classifying high-risk patients, and preventing diseases [12]. Research also indicates that integrating LLMs into clinical decision-making (CDM) training for medical students offers a cost-effective supplement to existing teaching methods [13]. Within the LLM framework, chatbots—an integral component of LLM technology—have also gained considerable attention. One of the most prominent chatbots, Chat Generative Pre-trained Transformer (ChatGPT), has been extensively studied for its applications in healthcare, such as ChatGPT 3.5 and GPT-4 [14,15]. In obstetrics and gynecology, researchers have explored its role in gynecologic oncology and genetic counseling. While ChatGPT may occasionally generate minor inaccuracies, studies indicate that it generally maintains a high level of diagnostic accuracy and comprehensiveness [16,17]. Furthermore, it serves as an effective tool for disseminating medical knowledge to the public, demonstrating substantial development potential and promising clinical applications [18].

China has made notable progress in the field of LLMs, developing several models, including Doubao and DeepSeek. Doubao has been evaluated for its performance in the Chinese dental licensing examination and the Chinese radiology attending physician qualification examination, demonstrating promising capabilities [19,20]. Another study demonstrates Doubao’s capability in traditional Chinese medicine diagnosis and treatment recommendations [21]. Recently, DeepSeek, a Chinese-developed LLM, has gained considerable attention in the AI research community [22,23,24]. With its exceptional overall performance and relatively low API costs, DeepSeek has generated considerable interest and been applied in various settings. Research has also highlighted DeepSeek’s powerful role in clinical decision-making [25]. Evaluations have suggested that the DeepSeek-V3 model can perform competitively against other advanced LLMs, such as Claude-3.5 and GPT-4o, in several domains. Additionally, DeepSeek-R1, released on 20 January 2025, is often discussed in comparative analyses as performing at a level comparable to leading models like OpenAI-o1. Its 32B and 70B models, distilled from DeepSeek-R1, have also demonstrated capabilities comparable to OpenAI o1-mini. These advancements have sparked considerable interest regarding their potential applications in the diagnosis and treatment of diseases. In fact, a study has also demonstrated DeepSeek’s high accuracy and robust chain-of-reasoning capabilities when answering open-ended questions about hip osteoarthritis from the American Academy of Orthopedic Surgeons (AAOS) [26]. Another study has shown that DeepSeek can effectively address common low back pain (LBP) issues and generate patient education materials [27]. However, these two LLMs have yet to be implemented in the diagnosis and treatment of ovarian cancer.

This study aims to assess the clinical utility of DeepSeek-R1 and Doubao-1.5-pro in ovarian cancer management by evaluating their diagnostic and therapeutic capabilities. The ultimate objective is to enhance decision-making in ovarian cancer treatment and improve healthcare services for patients.

## 2. Materials and Methods

To enable a more comprehensive and objective analysis, we summarized and extracted 20 key issues related to the diagnosis and treatment of ovarian cancer based on the NCCN, FIGO, and ESMO guidelines (Table 1). These issues were classified into four categories: “Risk Factors and Prevention”, “Surgical”, “Medical”, and “Surveillance”, with five questions in each category (Supplementary File S6: “questions.docx”). The questions were submitted to both DeepSeek-R1 and Doubao-1.5-pro for responses. To minimize interaction bias, each question in the two LLMs should be submitted independently in a single “New chat.” At the same time, based on authoritative guidelines such as FIGO and NCCN, the wording and format of each question are standardized (Table 2). Subsequently, the answers from both systems were anonymized and evaluated by five chief physicians specializing in gynecologic oncology. Each answer was rated on a scale from 1 to 10, based on its completeness and accuracy.

To assess the clinical applicability and effectiveness of answers provided by LLMs, a threshold score of 7 is established. This threshold was established by five chief physicians based on criteria including the accuracy of core diagnostic and treatment information, the completeness of key content, and whether it meets clinical reference requirements. Subsequent scoring was then conducted on this foundation. Both individual ratings from experts and the average score derived from the five experts’ evaluations of the same question are considered. Answers with a score above 7 are classified as “Excellent,” while those scoring 7 or below are categorized as “Not Excellent.”

To assess potential differences in the scores across the four categories for each LLM, the Kruskal–Wallis test was employed. A *p*-value greater than or equal to 0.05 suggests that the null hypothesis cannot be rejected, indicating no significant difference in the median scores among the groups. A *p*-value less than 0.05 indicates the rejection of the null hypothesis, implying that at least one group’s median score significantly differs from the others. A higher H value indicates a greater disparity between the groups, suggesting that at least one group’s scores differ substantially from those of the other groups.

After categorizing the questions for both LLMs into four groups, bar charts are used to present the average scores and standard deviations for each aspect. To assess whether significant differences exist between the scores of the two LLMs for the same aspect, the Mann–Whitney-Wilcoxon test is applied. A *p*-value less than 0.05 indicates rejection of the null hypothesis, suggesting a significant difference between the scores of the two LLMs. Conversely, a *p*-value greater than or equal to 0.05 implies that the null hypothesis cannot be rejected, indicating no significant difference between the scores.

Radar charts were also introduced to present the average scores for each question from both LLMs, providing a more intuitive comparison of the score differences between the two models.

Some key details of the models are shown in the table below (Table 3).

As this study does not involve human subjects, it is exempt from human subjects research regulations.

## 3. Results

This study presents 20 questions, and five experts independently evaluated the responses of two LLMs. In total, 200 scores were collected, with 100 scores assigned to each LLM. For DeepSeek-R1, 98 out of the 100 scores were rated as “Excellent,” while only 2 scores were rated as “Not Excellent,” accounting for 2%. In contrast, for Doubao-1.5-pro, only 41 out of the 100 scores received an “Excellent” rating, while 58 were classified as “Not Excellent,” representing 58% (Table 4 and Table 5). DeepSeek-R1’s scores are more concentrated in the 8–10 point range, with 9–10 points accounting for 83%. Doubao-1.5-pro, on the other hand, is more clustered in the 6–8 point range, reaching 65%. Both models exhibit a certain degree of clustering (Supplementary File S3: “deepseek scores.xlsx”; Supplementary File S4: “doubao scores.xlsx”).

Regarding the average scores for the 20 questions, all 20 responses from DeepSeek-R1 were rated as “Excellent.” In comparison, for Doubao-1.5-pro, only 9 responses were rated as “Excellent,” corresponding to questions Q1 to Q7, as well as Q10 and Q19 (Table 4 and Table 6).

The standard deviations for the 20 questions also varied. For DeepSeek-R1, the smallest standard deviation was observed for Q9, with a value of 0.00, while the largest was found for Q1 and Q8, both with a standard deviation of 1.10. In contrast, for Doubao-1.5-pro, the smallest standard deviations occurred for Q6 and Q18, both with a value of 0.55, while the largest was observed for Q3, with a standard deviation of 1.82 (Table 1 and Table 3).

The results of the Kruskal–Wallis Test for the two LLMs also differed. For DeepSeek-R1, the *p*-value was 0.07316 (>0.05), whereas for Doubao-1.5-pro, the *p*-value was 1.575 × 10^−5^ (≤0.05) (Table 7).

The scoring results for the four aspects also revealed significant differences. Each aspect consists of 5 questions, with 5 experts scoring each question, yielding a total of 25 scores per aspect. For DeepSeek-R1, both the “Risk Factors and Prevention” and “Surgical” aspects received 24 “Excellent” ratings, accounting for 96%. The “Medical” and “Surveillance” aspects achieved a 100% “Excellent” rating. In contrast, for Doubao-1.5-pro, the “Risk Factors and Prevention” aspect received only 17 “Excellent” ratings, or 68%; the “Surgical” aspect received 13 “Excellent” ratings, corresponding to 52%; the “Medical” aspect received just 3 “Excellent” ratings, representing 12%; and the “Surveillance” aspect received 8 “Excellent” ratings, accounting for 32% (Table 2).

**Figure 1:**
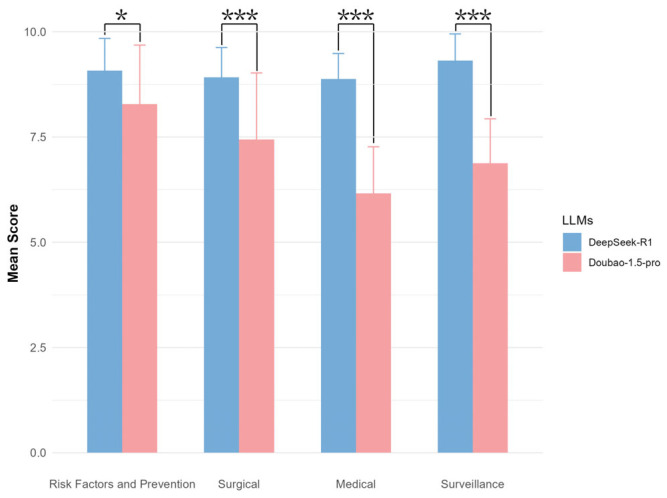
Comparative Performance Analysis of DeepSeek-R1 and Doubao-1.5-pro: Mean Expert Scores (±SD) in four categories for Ovarian Cancer. (1) “*” means *p* < 0.05; (2) “***” means *p* < 0.001.

A Mann–Whitney-Wilcoxon test was also conducted between the two LLMs for the four aspects. The *p*-values for all four aspects were found to be less than 0.05, with the *p*-value for the “Risk Factors and Prevention” aspect ranging from 0.01 to 0.05, and the *p*-values for the “Surgical,” “Medical,” and “Surveillance” aspects all being less than 0.001. The bar charts further illustrate that Doubao-1.5-pro scored lower than DeepSeek-R1 across all aspects, with the largest difference observed in the “Medical” aspect, followed by “Surveillance,” “Surgical,” and the smallest difference in “Risk Factors and Prevention.” (Figure 1).

**Figure 2:**
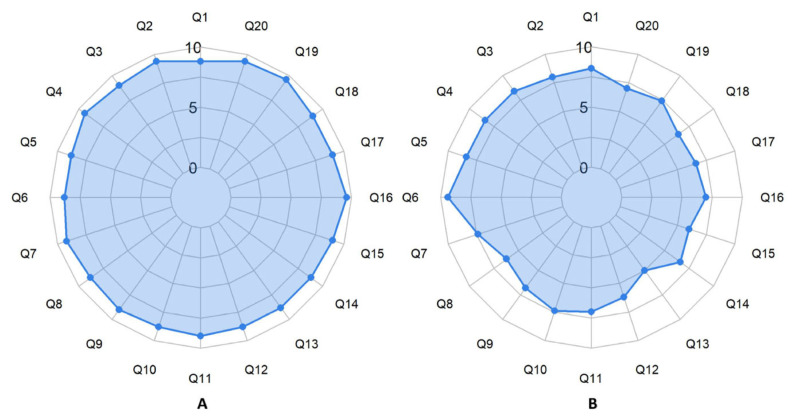
Comparison of Average Scores of 20 Questions between Two Large Language Models: Radar Chart. (**A**) The Average Scores of 20 Questions from DeepSeek-R1. (**B**) The Average Scores of 20 Questions from Doubao-1.5-pro.

The radar chart similarly reveals that, overall, Doubao-1.5-pro performs weaker than DeepSeek-R1. Only in Q6 did Doubao-1.5-pro outperform DeepSeek-R1, while in all other questions, DeepSeek-R1 demonstrated superior performance. The largest disparity was observed in Q15, where the average score difference between the two models reached its maximum value of 3 (Figure 2).

## 4. Discussion

Our research demonstrates that DeepSeek-R1 delivers excellent responses to a series of questions related to ovarian cancer, whereas Doubao-1.5-pro performs notably well in addressing risk factors and prevention of ovarian cancer. However, its performance in other areas is less remarkable, and Q15 serves as a prime example. The radar chart clearly demonstrates that DeepSeek-R1 significantly outperforms Doubao-1.5-pro on this question. This is primarily because DeepSeek-R1’s answers align more closely with the guidelines, feature more robust evidence-based support, contain more in-depth clinical specifics, and provide detailed explanations for certain medication data. In contrast, Doubao-1.5-pro’s responses are relatively superficial and lack key clinical details, remaining merely at the level of general medical popularization and thus failing to meet the requirements of professional clinical practice.

While existing research on ChatGPT is extensive and well-documented, studies specifically focused on DeepSeek-R1 remain limited. Current literature highlights its application in cardiopulmonary resuscitation (CPR) education [28], comparing various LLMs. These studies indicate that, while LLMs generally cover the essential points, they encounter challenges in addressing finer details, such as DeepSeek’s tendency to use overly technical terminology. This suggests that LLMs, including DeepSeek, are starting to make their mark in the medical field.

Furthermore, some studies have observed that DeepSeek-R1 demonstrates a more cautious and complex thought process than GPT-4 when responding to the same medical question. Its answers also exhibit a more critical approach [29]. This observation was also evident in our study. Specifically, after querying both LLMs, DeepSeek-R1 took noticeably longer to process and generate its response. As a result, the answers provided by DeepSeek-R1 were more detailed and logically coherent than those generated by Doubao-1.5-pro.

Surely, DeepSeek-R1 is not without its limitations. We identified several inaccuracies or omissions in its responses, particularly concerning details or overly generalized statements (Table 8). For example, in Q8, when asked about which ovarian cancer patients are eligible for fertility-sparing surgery, DeepSeek-R1 cited stage IA and some stage IC as appropriate indications. However, eligibility for fertility-sparing surgery varies depending on the histological type of ovarian cancer. For instance, patients with stage I malignant germ cell tumors (according to FIGO) are indeed eligible for fertility-sparing surgery. In Q9, regarding second cytoreductive surgery for platinum-sensitive recurrent patients, the response should have specified the absence of ascites as a key condition, but this was omitted. Meanwhile, in Q12, when discussing hyperthermic intraperitoneal chemotherapy (HIPEC) for ovarian cancer patients, DeepSeek-R1 indicated that the treatment was appropriate for “advanced ovarian cancer (FIGO stage III).” However, the latest NCCN guidelines only refer to “advanced ovarian cancer” without specifying a particular FIGO stage, making it overly prescriptive to limit it to stage III. In contrast, Doubao-1.5-pro scored lower and presented even more issues in its responses.

We believe that this issue primarily arises from the fact that the data used for training was collected too early and has not been updated promptly. This is a common challenge for many LLMs when applied in the medical field. For instance, when researchers evaluated ChatGPT’s capabilities in ovarian cancer diagnosis and treatment, their findings also pointed out that some issues were due to outdated data [16]. In conclusion, multiple studies have shown that for LLMs to be more effectively applied in the medical field, the timely collection, training, application, and updating of relevant data and materials are essential. Actually, other research has further highlighted the limitations of LLMs, including DeepSeek, in clinical problem-solving due to inflexible reasoning [30]. A study also notes that while DeepSeek handles common LBP problems effectively, the content it generates still exhibits significant issues in terms of readability, accuracy, and hallucinations [27]. Regarding DeepSeek-R1’s current state—“generally correct answers with minor issues in details”—we maintain that human medical professionals must still lead the implementation of medical practices. Over-reliance on LLM outputs in clinical settings may give rise to inappropriate diagnostic and therapeutic decisions due to such inherent limitations as minor detail inaccuracies and outdated training data, thus posing potential risks to patient safety and clinical practice quality.

Moreover, while the “critical thinking” demonstrated by LLMs should be acknowledged, their responses are often excessively detailed. Compared to human doctors, LLMs’ answers lack a humanistic touch. This can burden non-experts who attempt to understand and apply the knowledge. Many LLM users are non-medical professionals, and their primary intent is often consultation. These long-winded, overly detailed, and impersonal responses can easily lead to comprehension difficulties.

Additionally, we observed a significant disparity in the results between DeepSeek-R1 and Doubao-1.5-pro. Such differences may plausibly be associated with several potential factors. First, DeepSeek-R1 may be trained on a larger dataset than Doubao-1.5-pro, allowing it to leverage more comprehensive data. Second, the optimization priorities of the two LLMs likely differ. DeepSeek-R1 may have an advantage in the ovarian cancer domain, integrating more specialized ovarian cancer-related knowledge and a more advanced medical knowledge graph. Furthermore, DeepSeek-R1’s reasoning depth may surpass that of Doubao-1.5-pro, enabling it to generate more comprehensive and nuanced answers. Additionally, the default settings of the two models may not be entirely consistent, potentially leading to differences in generated responses regarding aspects such as text length and content precision. For non-AI professionals, including medical personnel, default settings are typically the go-to option. Consequently, they often overlook or are unable to adjust relevant parameters, resulting in less-than-ideal outcomes. These findings suggest that when applying LLMs to address medical issues, selecting the appropriate LLM is crucial for obtaining more accurate and relevant results, particularly for non-AI practitioners who lack expertise in adjusting parameter settings. Concomitantly, researchers have recently posed inquiries regarding the selective utilization of LLMs. This further underscores the imperative for employing distinct LLMs for disparate tasks [31].

In future research, we plan to explore the capabilities of LLMs further. First, we will submit the same question to LLMs multiple times (e.g., 10 times) and evaluate the content they produce. By analyzing the scores and their fluctuations, we can delve into the stability of content generation in LLMs represented by DeepSeek and Doubao. Additionally, we can ask the same question using different phrasing styles, such as varying word choices, sentence structures, colloquial expressions, and elliptical questions. Analyzing how LLMs respond to different phrasing styles will allow us to assess their ability to adapt to more complex and dynamic clinical application scenarios and meet the diverse needs of clinical work. In addition, we can further incorporate leading international models such as GPT-4o and Claude into our research to deepen our findings. Moreover, a comparative study could also be conducted involving gynecologic oncologists with varying years of experience as controls, analyzing the diagnostic and therapeutic efficacy of LLM versus human physicians to assess the gaps between LLM and human doctors across different clinical scenarios. We also plan to introduce real clinical ovarian cancer cases to evaluate the models’ ability to analyze and solve practical clinical problems. We also intend to optimize and evaluate Chinese LLMs by constructing retrieval-augmented and guideline-grounded systems in subsequent research, aiming to further enhance the faithfulness of clinical question answering and reduce the occurrence of hallucinations. And future research could adopt validated multidimensional assessment rubrics, separately evaluating core dimensions such as accuracy, guideline concordance, and safety-related completeness. This will help dissect specific failure modes of LLMs and enhance the clinical interpretability of evaluation results.

There are some limitations to this study. In the high-risk field of ovarian cancer management, conducting systematic safety and harm assessments of large language models is crucial, yet this represents a limitation of the present study. Based on relevant clinical guidelines, we conducted a retrospective review of responses from both models. We confirmed that DeepSeek-R1 did not generate explicit contraindications or harmful clinical recommendations, with only non-critical omissions of details. Similarly, Doubao-1.5-pro did not provide explicit harmful recommendations, but some responses omitted high-risk critical clinical decision-making information, such as surgical indications and drug eligibility criteria. We contend that systematic safety assessment should become a core evaluation dimension in future research. The threshold of 7/10, while based on expert consensus, inevitably carries a degree of subjectivity. The sample size is relatively small, consisting of only 20 questions and two LLMs, evaluated by just five experts. 20 questions may be insufficient to provide a detailed description of ovarian cancer diagnosis and treatment. Therefore, increasing the number of ovarian cancer diagnoses and treatments is one of the directions for future research. Additionally, all five experts are from the same institution, which may have influenced the homogeneity of the responses and introduced potential evaluator bias. Future multi-center evaluations with raters from diverse geographic and institutional backgrounds would strengthen generalizability. Furthermore, the model responses were evaluated based on a single, standardized prompt format; performance may vary with different phrasings of the same clinical question. Finally, this study provides a static evaluation of the models’ knowledge at a fixed point in time and did not assess their performance within dynamic clinical workflows.

The strength of this study lies in its pioneering exploration of DeepSeek and Doubao’s applications in ovarian cancer diagnosis and treatment. It offers a comprehensive, multi-angle evaluation of both models’ latest versions in relation to various aspects of ovarian cancer while comparing the two LLMs and highlighting the functionalities of DeepSeek.

## 5. Conclusions

In conclusion, selecting the appropriate LLM for medical applications is critical. DeepSeek, particularly the DeepSeek-R1 version, shows certain potential in the field of ovarian cancer diagnosis and treatment. It has demonstrated strong performance in areas such as risk factors and prevention, surgery, treatment, and postoperative care. However, it still generates occasional minor inaccuracies in clinical details, and it lacks sufficient humanistic elements. Currently, DeepSeek-R1 can serve as a supplementary tool for medical education and clinical assistive diagnosis, and its use must be under the direct guidance and supervision of qualified gynecologic oncology clinicians. Importantly, it remains unfit for independent involvement in any full-scale ovarian cancer diagnostic or therapeutic decision-making processes. We recommend that developers continue to refine and update the training materials to enhance the model’s applicability and benefit to a wider population.

## Figures and Tables

**Table 1:** The Question Set.

Category	Number	Question
Risk Factors and Prevention	Q1	What are the risk factors for ovarian cancer?
	Q2	What genetic and molecular testing is recommended in patients with ovarian cancer?
	Q3	How much do BRCA mutations increase risk for ovarian cancer?
	Q4	Who should be tested for BRCA mutations?
	Q5	How can you prevent cancer if you test positive for BRCA mutations?
Surgical	Q6	What are the principles of surgery for newly diagnosed invasive epithelial ovarian cancer involving the pelvis and upper abdomen (stage ≥ IIB)?
	Q7	What is the surgical protocol for risk-reducing salpingo-oophorectomy?
	Q8	Can fertility-sparing surgery be used in patients with ovarian cancer?
	Q9	When should secondary cytoreduction be considered in patients with ovarian cancer?
	Q10	Should ovarian cancer surgery be performed using minimally invasive techniques or laparotomy?
Medical	Q11	Which patients with ovarian cancer should receive neoadjuvant chemotherapy?
	Q12	When should HIPEC be used in ovarian cancer?
	Q13	Which patients with ovarian cancer should receive bevacizumab for maintenance therapy?
	Q14	Should patients with recurrent epithelial ovarian cancer who had received one prior PARP inhibitor as maintenance therapy be retreated with a PARP inhibitor?
	Q15	Should patients with ovarian cancer be treated with immunotherapy or not?
Surveillance	Q16	How should patients with ovarian cancer be followed after treatment is complete?
	Q17	How to define platinum-resistant recurrent ovarian cancer?
	Q18	What is the management strategy for biochemical relapse of ovarian cancer?
	Q19	What are the independent prognostic factors for ovarian cancer?
	Q20	How to manage chemotherapy-associated adverse events in patients with ovarian cancer?

**Table 2:** The Representative Responses of DeepSeek-R1 and Doubao-1.5-pro.

LLMs	Number	Representative Responses
DeepSeek-R1	Q16	1. Follow-Up Objectives Detect recurrence: Early identification of asymptomatic relapse for timely intervention. Manage treatment-related complications: Address chemotherapy-induced neuropathy, myelosuppression, or surgical sequelae. Improve quality of life: Focus on psychological health, nutrition, bone health, and sexual function. 2. Follow-Up Schedule and Components First 2 years: Every 3–4 months: History and physical exam: Assess symptoms (bloating, pain, weight loss), pelvic examination. Tumor markers: CA-125 (if elevated pretreatment), HE4 (optional). Imaging: Ordered only for symptoms, rising CA-125, or clinical suspicion (e.g., contrast CT/MRI). Years 3–5: Every 4–6 months: Same as above, with reduced imaging frequency. Beyond 5 years: Annually: Focus on long-term complications (cardiovascular disease, osteoporosis, secondary cancers). 3. Recurrence Monitoring Symptom alerts: New/worsening ascites, bowel obstruction, pelvic mass, dyspnea (pleural effusion), or systemic decline. CA-125 trends: Persistent elevation (≥2× ULN) warrants evaluation, excluding non-malignant causes. Asymptomatic slow rise may be observed without immediate intervention. Imaging confirmation: CT/MRI findings require PET-CT for systemic staging. Biopsy suspicious lesions for pathology and molecular profiling (e.g., BRCA, HRD). 4. Long-Term Complication Management Menopausal symptoms: Hormone replacement therapy (HRT) individualized (caution in BRCA carriers). Non-hormonal options (e.g., SSRIs) for vasomotor symptoms. Bone health: Annual DEXA scans, calcium (1200 mg/day), and vitamin D (800 IU/day). Bisphosphonates (e.g., zoledronate) or denosumab for osteoporosis prevention. Cardiovascular risk: Monitor blood pressure, lipids, glucose; promote smoking cessation, exercise, and Mediterranean diet. 5. Psychosocial Support and Quality of Life Mental health screening: Refer to psychologists/support groups for anxiety/depression. Nutrition counseling: Avoid obesity (BMI > 30 increases recurrence risk); emphasize plant-based protein and fiber. Sexual health: Topical estrogen for vaginal dryness; lubricants for dyspareunia. Fertility counseling if applicable. 6. Genetic Counseling and Family Screening Genetic testing: Recommend BRCA1/2, HRD, and other ovarian cancer genes (e.g., RAD51C/D) if not previously done. Family risk assessment: First-degree relatives require genetic counseling and screening (e.g., TVUS + CA-125). 7. Patient Education Symptom diary: Document bloating, urinary frequency, fatigue, and report promptly. Lifestyle modifications: Avoid smoking, limit alcohol, maintain moderate exercise (150 min/week). Follow-up adherence: Stress the importance of regular visits for early detection.
Doubao-1.5-pro	Q4	People with a family history of ovarian cancer: If there are ovarian cancer patients in the family, especially if first-degree relatives (such as mothers, sisters, daughters) have ovarian cancer, then other family members should consider undergoing BRCA gene mutation testing, as there may be an increased risk of disease due to genetic factors. People with a family history of breast cancer: If there are multiple members with breast cancer in the family, especially if the age of onset is early (such as under 50 years old), or if there are male breast cancer patients, it is necessary for their relatives to undergo BRCA gene mutation testing, because BRCA gene mutations are closely related to the occurrence of both breast cancer and ovarian cancer. Patients with ovarian cancer: For patients who have been diagnosed with ovarian cancer, testing for BRCA gene mutations helps to understand the pathogenesis of the tumor, guide the selection of subsequent treatment plans, such as whether they are suitable for targeted drugs such as PARP inhibitors, and also assess the disease risk of family members. Patients with breast cancer: Especially patients with triple-negative breast cancer or those with an early age of onset, undergoing BRCA gene mutation testing can help determine whether there is a genetic cause of the disease, which is of great significance for subsequent treatment and family genetic risk assessment. People with a family history of other related hereditary cancer syndromes: If there are patients with hereditary cancer syndromes related to BRCA gene mutations in the family, such as Lynch syndrome and Li-Fraumeni syndrome, other family members should also consider undergoing testing.

**Table 3:** The Key Details of the Models.

Details	DeepSeek	Doubao
Model versioning	DeepSeek-R1	Doubao-1.5-pro
Temperature	0.60	0.70
Max tokens	32K	12K
Top-p	0.95	0.90
Random seed	Official default	Official default
System prompt	Official default	Official default

**Table 4:** Descriptive Statistics of DeepSeek-R1’s Answer Quality Evaluation.

Question	Mean	SD	Min	Max	Performance
Q1	8.80	1.10	7.00	10.00	Excellent
Q2	9.40	0.89	8.00	10.00	Excellent
Q3	9.00	0.71	8.00	10.00	Excellent
Q4	9.40	0.55	9.00	10.00	Excellent
Q5	8.80	0.45	8.00	9.00	Excellent
Q6	8.80	0.45	8.00	9.00	Excellent
Q7	9.20	0.84	8.00	10.00	Excellent
Q8	8.80	1.10	7.00	10.00	Excellent
Q9	9.00	0.00	9.00	9.00	Excellent
Q10	8.80	0.84	8.00	10.00	Excellent
Q11	9.00	0.71	8.00	10.00	Excellent
Q12	8.80	0.45	8.00	9.00	Excellent
Q13	8.80	0.84	8.00	10.00	Excellent
Q14	8.80	0.45	8.00	9.00	Excellent
Q15	9.00	0.71	8.00	10.00	Excellent
Q16	9.60	0.55	9.00	10.00	Excellent
Q17	9.00	0.71	8.00	10.00	Excellent
Q18	9.00	0.71	8.00	10.00	Excellent
Q19	9.60	0.55	9.00	10.00	Excellent
Q20	9.40	0.55	9.00	10.00	Excellent

**Table 5:** Specific Statistics of Answer Performance from DeepSeek-R1 and Doubao-1.5-pro.

Aspects	DeepSeek-R1		Doubao-1.5-Pro	
	Excellent (%)	Not Excellent (%)	Excellent (%)	Not Excellent (%)
Risk Factors and Prevention (5 questions × 5 doctors, *n* = 25)	24 (96.00)	1 (4.00)	17 (68.00)	8 (32.00)
Surgical (5 questions × 5 doctors, *n* = 25)	24 (96.00)	1 (4.00)	13 (52.00)	12 (48.00)
Medical (5 questions × 5 doctors, *n* = 25)	25 (100.00)	0(0)	3 (12.00)	22 (88.00)
Surveillance (5 questions × 5 doctors, *n* = 25)	25 (100.00)	0(0)	8 (32.00)	17 (68.00)

**Table 6:** Descriptive Statistics of Doubao-1.5-pro’s Answer Quality Evaluation.

Question	Mean	SD	Min	Max	Performance
Q1	8.20	1.48	6.00	10.00	Excellent
Q2	8.00	1.41	6.00	9.00	Excellent
Q3	8.40	1.82	6.00	10.00	Excellent
Q4	8.40	1.34	7.00	10.00	Excellent
Q5	8.40	1.52	6.00	10.00	Excellent
Q6	9.40	0.55	9.00	10.00	Excellent
Q7	7.40	1.67	5.00	9.00	Excellent
Q8	6.20	1.10	5.00	8.00	NOT Excellent
Q9	6.80	1.30	5.00	8.00	NOT Excellent
Q10	7.40	1.34	6.00	9.00	Excellent
Q11	7.00	0.71	6.00	8.00	NOT Excellent
Q12	6.20	0.84	5.00	7.00	NOT Excellent
Q13	5.00	0.71	4.00	6.00	NOT Excellent
Q14	6.60	1.34	5.00	8.00	NOT Excellent
Q15	6.00	1.00	5.00	7.00	NOT Excellent
Q16	7.00	1.00	6.00	8.00	NOT Excellent
Q17	6.60	1.67	4.00	8.00	NOT Excellent
Q18	6.40	0.55	6.00	7.00	NOT Excellent
Q19	7.40	1.14	6.00	9.00	Excellent
Q20	7.00	0.71	6.00	8.00	NOT Excellent

**Table 7:** Kruskal–Wallis Test of Answers from DeepSeek-R1 and Doubao-1.5-pro (classified into four categories).

LLMs	k	df	Kruskal–Wallis Chi-Squared	*p*-Value
DeepSeek-R1	4	3	6.9607	0.07316 (>0.05)
Doubao-1.5-pro	4	3	24.959	1.575 × 10^−5^ (≤0.05)

**Table 8:** Examples of Inaccuracies or Omissions in DeepSeek-R1’s Answers.

Question	Inaccurate Statement by DeepSeek-R1	Correct Guideline Interpretation
Q8	All patients with FIGO stage IA and some with stage IC ovarian cancer are eligible for fertility-sparing surgery.	Eligibility for fertility-sparing surgery depends on histological type. For example, patients with stage I malignant germ cell tumors (per FIGO) are eligible, while epithelial cancers require stricter criteria (e.g., low-risk IA).
Q9	Secondary cytoreductive surgery is suitable for platinum-sensitive recurrent ovarian cancer patients.	Secondary cytoreductive surgery requires specific conditions, including absence of ascites, which was omitted in the response.
Q12	Hyperthermic intraperitoneal chemotherapy (HIPEC) is indicated for “advanced ovarian cancer (FIGO stage III)”.	Current NCCN guidelines (2024 v3) refer to “advanced ovarian cancer” without specifying a FIGO stage. Limiting the indication to Stage III is arbitrary.

## Data Availability

The data presented in this study are available on request from the corresponding authors. The data are not publicly available due to privacy and confidentiality considerations.

## Supplementary Materials

The following supporting information can be downloaded at: https://www.mdpi.com/article/10.3390/diagnostics16040616/s1, Supplementary File S1: Answers from DeepSeek.docx; Supplementary File S2: Answers from Doubao.docx; Supplementary File S3: deepseek scores.xlsx; Supplementary File S4: doubao scores.xlsx; Supplementary File S5: *p*-value of Mann–Whitney U test for 4 aspects.xlsx; Supplementary File S6: questions.docx.

## Abbreviations

The following abbreviations are used in this manuscript:

AI	Artificial Intelligence
LLM	Large Language Model
CDM	Clinical Decision-making
ChatGPT	Chat Generative Pre-trained Transformer
AAOS	American Academy of Orthopedic Surgeons
LBP	Low Back Pain
HRT	Hormone replacement therapy
CPR	Cardiopulmonary Resuscitation
HIPEC	Hyperthermic Intraperitoneal Chemotherapy
