# Supplementary material for: Decoding AI Competence: Benchmarking Large Language Models (LLMs) in Ovarian Cancer Diagnosis and Treatment—A Systematic Evaluation of Generative AI Accuracy and Completeness
Source: Diagnostics (Basel). 2026 Feb 20;16(4):616. doi: 10.3390/diagnostics16040616 (PMC12939671; doi:10.3390/diagnostics16040616)
Supplement: Supplementary file 1 [file diagnostics-16-00616-s001.zip › FileS1-Answers from DeepSeek.pdf]

Risk Factors and Prevention:

1.What are the risk factors for ovarian cancer?

Risk factors for ovarian cancer include:

Age: Risk increases with age, most cases occur after menopause (over 55 years).

Genetic factors:

①BRCA1/BRCA2 gene mutations (linked to breast and ovarian cancers).

②Hereditary syndromes like Lynch syndrome.

③Family history: First-degree relatives (mother, sister, daughter) with ovarian, breast, or colorectal cancer.

Reproductive and hormonal factors:

①Nulliparity (no pregnancies) or fewer pregnancies.

②Early menarche (before age 12) or late menopause (after 52).

③Long-term estrogen-only hormone replacement therapy.

Contraceptive use: Long-term oral contraceptive use may reduce risk, while non-users face higher risk.

Endometriosis: Increases risk of clear cell or endometrioid ovarian cancers.

Obesity: High BMI may mildly elevate risk.

Lifestyle: Smoking may raise risk of mucinous ovarian cancer.

Fertility treatments: Some studies suggest ovulation-stimulating drugs may play a role, but evidence is inconclusive.

Ethnicity: Higher incidence in White women compared to other groups.

Note: Having risk factors does not guarantee cancer, and some risks (e.g., genetic mutations) can be mitigated through preventive measures (e.g., surgery).

2.What genetic and molecular testing is recommended in patients with ovarian cancer?

Recommended genetic and molecular testing for ovarian cancer patients includes:

①BRCA1/BRCA2 Testing:

Significance: Guides PARP inhibitor use (e.g., olaparib) and assesses hereditary cancer risk.

Samples: Tumor tissue (somatic mutations) or blood/saliva (germline mutations).

②Homologous Recombination Deficiency (HRD) Testing:

Purpose: Evaluates HRD status (including BRCA mutations and other homologous recombination gene defects).

Methods: Genomic instability score (e.g., Myriad myChoice® CDx) or gene panel testing.

Clinical Use: Predicts PARP inhibitor efficacy (greater benefit in HRD-positive tumors).

③Mismatch Repair (MMR)/Microsatellite Instability (MSI) Testing:

Significance: Screens for Lynch syndrome and identifies candidates for immunotherapy (e.g., pembrolizumab).

Methods: Immunohistochemistry (IHC) for MMR proteins (MLH1, MSH2, MSH6, PMS2) or MSI molecular testing.

④Somatic Mutation Analysis (e.g., TP53, KRAS, PIK3CA):

Purpose: Identifies therapeutic targets or prognostic markers (e.g., TP53 mutations in high-grade serous carcinoma).

⑤HER2/neu Amplification Testing:

Application: May guide anti-HER2 therapy in rare subtypes (e.g., mucinous ovarian cancer).

⑥NTRK Gene Fusion Testing:

Significance: Rare cases may benefit from TRK inhibitors (e.g., larotrectinib).

⑦Tumor Mutational Burden (TMB) and PD-L1 Expression:

Use: Assesses potential immunotherapy response, though evidence in ovarian cancer is limited.

Key Considerations:

Genetic Counseling: Required if germline mutations (e.g., BRCA1/2) are detected.

Tissue Preference: Tumor tissue is preferred over blood to avoid missing somatic mutations.

Guideline References: NCCN and ESMO recommend BRCA/HRD testing for all epithelial ovarian cancers to inform treatment and hereditary risk management.

3.How much do BRCA mutations increase risk for ovarian cancer?

BRCA1 and BRCA2 mutations significantly increase the risk of ovarian cancer:

BRCA1 Mutation: Lifetime ovarian cancer risk is approximately 35%-70% (vs. 1%-2% in the general population).

BRCA2 Mutation: The risk is slightly lower, around 10%-30%.

Mechanism: These genes encode proteins involved in DNA repair (e.g., homologous recombination). Mutations impair this function, leading to genomic instability and cancer development.

Other Factors: Risk may vary based on family history, specific mutation type, or coexisting genetic variants.

Management: High-risk individuals may opt for regular screening, risk-reducing surgery (e.g., salpingo-oophorectomy), or targeted therapies (e.g., PARP inhibitors).

4.Who should be tested for BRCA mutations?

The following individuals should consider BRCA1/BRCA2 genetic testing:

①Personal or Family Cancer History:

Diagnosis of breast cancer (especially early-onset ≤45 years), ovarian/fallopian tube/peritoneal cancer, or high-risk/metastatic prostate cancer.

Family history of multiple related cancers or individuals with multiple primary cancers.

②Specific Ethnic Background:

Ashkenazi Jewish ancestry (≈1/40 carry BRCA mutations vs. 1/400 in the general population).

③Known Family Mutation:

Relatives with confirmed pathogenic BRCA mutations.

④Other High-Risk Features:

Triple-negative breast cancer (TNBC, particularly if diagnosed ≤60 years).

Male breast cancer patients.

⑤Preventive Management:

Those considering risk-reducing surgery (e.g., prophylactic oophorectomy) or requiring guidance for targeted therapies (e.g., PARP inhibitors).

⑥Genetic Counseling:

Pre-test genetic counseling is mandatory to assess risk, interpret results, and plan clinical management.

5.How can you prevent cancer if you test positive for BRCA mutations?

BRCA mutation carriers can reduce cancer risk through the following strategies:

①Risk-Reducing Surgery:

Bilateral Salpingo-Oophorectomy (RRSO): Recommended at 35-40 for BRCA1 and 40-45 for BRCA2 carriers, reducing ovarian cancer risk by 90%-95% and breast cancer risk by 50% (premenopausal women).

Bilateral Mastectomy: Reduces breast cancer risk by 90%-95%, suitable for high-risk individuals avoiding long-term screening.

②Enhanced Surveillance:

Breast Cancer: Annual breast MRI from age 25 (preferred) and mammograms (combined after 40).

Ovarian Cancer: Transvaginal ultrasound and CA-125 testing (limited efficacy, mainly interim monitoring before surgery).

③Pharmacologic Prevention:

PARP Inhibitors (e.g., Olaparib): Used for maintenance therapy in cancer patients; preventive use in healthy carriers is under clinical trials.

Tamoxifen/Raloxifene: Reduce breast cancer risk by 50% but require evaluation of side effects (e.g., thrombosis, endometrial cancer).

④Lifestyle Modifications:

Avoid smoking, limit alcohol, maintain healthy weight to potentially lower risk.

⑤Reproductive & Hormonal Management:

Complete childbearing before RRSO; short-term hormone replacement therapy may be considered post-surgery (individualized assessment).

⑥Genetic Counseling & Support:

Regular follow-ups, support groups, and dynamic risk management planning.

Surgical:

6.What are the principles of surgery for newly diagnosed invasive epithelial ovarian cancer involving the pelvis and upper abdomen (stage ≥IIB)?

The surgical principles for newly diagnosed invasive epithelial ovarian cancer involving the pelvis and upper abdomen (stage ⩾IIB) focus on **maximal cytoreductive surgery**, with key aspects including:

①Surgical Goals:

Optimal Debulking: Aim for residual disease <1 cm (ideally no macroscopic residue/R0). Smaller residual tumor burden correlates with improved survival.

②Surgical Extent:

Standard Procedures: Total hysterectomy + bilateral salpingo-oophorectomy + omentectomy + peritoneal biopsies/resection ± appendectomy.

Extended Resection: May require bowel resection, splenectomy, partial hepatectomy, diaphragmatic stripping, or lymph node dissection based on tumor spread.

Lymphadenectomy: Pelvic and para-aortic lymph node dissection (for accurate staging, especially if metastasis is suspected).

③Staging & Evaluation:

Comprehensive Staging: Thorough exploration of the upper abdomen (diaphragm, liver surface, splenic region, mesentery, etc.) and documentation of disease distribution.

Intraoperative Frozen Section: Used to confirm histology and extent when necessary.

④Multidisciplinary Team (MDT):

Collaboration among gynecologic oncologists, general surgeons, urologists, and hepatobiliary surgeons for complex cases (e.g., bowel obstruction, liver metastases).

⑤Integration with Adjuvant Therapy:

Postoperative platinum-based chemotherapy (e.g., paclitaxel + carboplatin), with possible addition of bevacizumab or PARP inhibitors (for BRCA-mutated cases).

Neoadjuvant Chemotherapy + Interval Cytoreduction if primary optimal debulking is unachievable.

⑥Special Considerations:

Fertility Preservation: Limited to highly selected young patients with early-stage disease (generally not applicable for ≥IIB).

Preoperative Assessment: Imaging (CT/MRI/PET-CT) and CA-125 to evaluate resectability.

7.What is the surgical protocol for risk-reducing salpingo-oophorectomy?

Surgical Protocol for Risk-Reducing Salpingo-Oophorectomy (RRSO)

①Indications

High-risk individuals: Carriers of BRCA1/BRCA2 mutations, Lynch syndrome, other hereditary ovarian/breast cancer syndromes, or significant family history.

Recommended ages:

BRCA1: 35-40 years (after childbearing).

BRCA2: 40-45 years (due to later ovarian cancer onset).

Lynch syndrome: 40-45 years or individualized.

②Preoperative Evaluation

Genetic confirmation: Pathogenic mutation verification.

Imaging: Pelvic ultrasound/MRI to exclude existing lesions.

Tumor markers: e.g., CA-125 (if elevated, further assessment required).

Multidisciplinary consultation: Involving gynecologic oncologists, genetic counselors, and psychologists.

③Surgical Procedure

Laparoscopic approach (preferred) or laparotomy.

Key steps:

1) Complete abdominal exploration to rule out occult tumors.
2) Bilateral removal of fallopian tubes and ovaries (avoid residual tissue).
3) Specimen retrieval via endoscopic bag to prevent tumor spread.
4) Intraoperative frozen section (if suspicious lesions are found).

④Postoperative Management

Pathology: Serial sectioning of tubes/ovaries with focus on serous tubal intraepithelial carcinoma (STIC).

Hormone Replacement Therapy (HRT):

Short-term low-dose HRT for premenopausal women (if no breast cancer contraindications).

Caution in BRCA carriers (potential breast cancer risk).

Complication management: Bleeding, infection, menopausal symptoms (e.g., hot flashes, osteoporosis).

⑤Follow-Up

Short-term: Monitor surgical complications (1-2 weeks).

Long-term:

Breast cancer screening (MRI/mammogram).

Bone health (DEXA scans, calcium/vitamin D supplementation).

Psychological support (menopause and cancer-related anxiety).

⑥Patient Counseling

Fertility and endocrine effects: Irreversible procedure; discuss fertility preservation (e.g., egg freezing) preoperatively.

Cancer risk: RRSO reduces ovarian cancer risk by 80%-90%, but residual primary peritoneal cancer risk remains (1%-2%).

8.Can fertility-sparing surgery be used in patients with ovarian cancer?

Can Fertility-Sparing Surgery Be Used in Patients with Ovarian Cancer?

①Indications

Pathology:

Germ cell tumors (e.g., dysgerminoma, immature teratoma): First-line option.

Sex cord-stromal tumors (e.g., granulosa cell tumors).

Early-stage epithelial ovarian cancer (limited to low-grade serous, mucinous, or endometrioid carcinoma).

Stage Criteria:

Stage IA (tumor confined to one ovary, intact capsule, no ascites).

Selected IC cases (IC1/IC2, intraoperative rupture/surface involvement without malignant ascites).

Patient Factors:

Young age with strong fertility desire.

Absence of hereditary cancer risks (e.g., no BRCA mutations).

②Contraindications

Advanced-stage disease (Stage II or higher).

High-risk histologies (e.g., high-grade serous carcinoma, clear cell carcinoma).

Bilateral ovarian or uterine involvement.

Hereditary cancer syndromes (e.g., Lynch syndrome, BRCA mutations).

③Surgical Approach

Standard Procedure:

Unilateral salpingo-oophorectomy (preserve uterus and contralateral ovary).

Comprehensive staging: Peritoneal biopsies, omentectomy, pelvic/para-aortic lymph node sampling.

Peritoneal washings for cytology.

Key Considerations:

Intraoperative frozen section to exclude occult metastasis.

Biopsy contralateral ovary if abnormal appearance.

④Postoperative Management

Adjuvant Therapy:

Germ cell tumors: Chemotherapy (e.g., BEP regimen) without removing residual ovary.

Epithelial cancer: Observation for IA G1; chemotherapy (platinum-based) for IA G2-3 or IC.

Fertility Timing: Recommend conception within 6-12 months post-treatment.

Follow-Up:

Pelvic ultrasound and tumor markers (e.g., CA-125, AFP, hCG) every 3-6 months.

Recommend ovarian removal after childbearing (especially for epithelial cancer).

⑤Prognosis and Risks

Survival: 5-year survival rates match radical surgery for early-stage disease (＞90% for IA).

Recurrence:

Epithelial cancer: 10%-15% recurrence, often in contralateral ovary or peritoneum.

Germ cell tumors: ＜5% recurrence, highly chemo-sensitive.

⑥Patient Counseling

Disclose recurrence risks and potential need for secondary surgery.

Recommend fertility preservation (e.g., egg/embryo freezing).

Emphasize lifelong surveillance.

9.When should secondary cytoreduction be considered in patients with ovarian cancer?

①Indications

Platinum-sensitive recurrence: Relapse ≥6 months after last chemotherapy (especially platinum-free interval ≥12 months).

Resectable disease:

Imaging (PET-CT/contrast CT/MRI) shows localized lesions (e.g., isolated masses, limited peritoneal metastases).

Anticipated R0 (no visible residue) or R1 (microscopic residue) resection.

Patient status:

Good performance status (ECOG 0-1).

No severe comorbidities (e.g., cardiopulmonary dysfunction).

②Contraindications

Platinum-resistant recurrence (relapse <6 months post-chemotherapy).

Extensive metastases (e.g., multiple liver lesions, diffuse peritoneal carcinomatosis, unresectable mesenteric masses).

Significant ascites/pleural effusion (indicative of disseminated disease).

Patient unfit for surgery or declining aggressive intervention.

③Preoperative Evaluation

Imaging:

PET-CT or contrast MRI/CT to map disease extent and organ involvement.

Diagnostic laparoscopy if resectability is uncertain.

Lab tests: CA-125 trends, hematologic/biochemical profiles.

Multidisciplinary review: Collaboration among gynecologic oncologists, radiologists, and surgeons.

④Surgical Goals and Extent

Objective: Achieve R0 resection (complete macroscopic removal).

Procedures:

Resection of recurrent lesions (e.g., pelvic masses, peritoneal nodules).

Selective lymphadenectomy (if nodal involvement suspected).

Bowel resection/anastomosis, splenectomy, or partial hepatectomy as needed.

⑤Postoperative Management

Adjuvant therapy:

Platinum-based chemotherapy (e.g., carboplatin + paclitaxel).

Targeted therapy (e.g., PARP inhibitors for BRCA mutants).

Complication monitoring: Bowel obstruction, anastomotic leak, infection.

Follow-up: Imaging and CA-125 every 3 months.

⑥Prognostic Factors

Favorable factors:

R0 resection, long platinum-free interval (≥18 months), ≤3 recurrent lesions.

Survival data:

Median survival of 50-60 months with R0 resection vs. significantly shorter survival with residual disease.

⑦Patient Counseling

Clarify risks (surgical complications) vs. benefits (prolonged survival).

Emphasize adherence to systemic therapy and lifelong surveillance.

10.Should ovarian cancer surgery be performed using minimally invasive techniques or laparotomy?

①Indications for Approach Selection

Minimally Invasive Surgery (MIS; Laparoscopy/Robotic):

Early-stage disease (Stage I): Tumor confined to one ovary with intact capsule (IA-IC1).

No preoperative evidence of metastasis: Imaging (CT/MRI) excludes lymph node or peritoneal spread.

Low-risk histologies: Borderline tumors, low-grade epithelial cancers, select germ cell tumors.

Specific patient needs: Obesity or cardiopulmonary comorbidities requiring reduced trauma.

Laparotomy:

Advanced disease (Stage II-IV): Requires comprehensive exploration and maximal cytoreduction (aiming for R0).

Suspected metastasis: Imaging suggests peritoneal carcinomatosis, lymphadenopathy, or omental involvement.

High-grade serous carcinoma: High risk of occult metastases, necessitating complete staging.

Intraoperative conversion: Switch to laparotomy if MIS reveals unexpected disease extent.

②Pros and Cons of MIS

Advantages:

Less trauma, blood loss, faster recovery, shorter hospitalization.

Reduced postoperative pain and adhesion risk.

Risks/Limitations:

Limited visualization (risk of missing occult lesions, leading to understaging).

Tumor rupture/spillage risk (mandates strict use of retrieval bags).

Requires high surgical expertise and steep learning curve.

③Pros and Cons of Laparotomy

Advantages:

Complete abdominal exploration for accurate staging.

Higher likelihood of optimal cytoreduction in complex cases.

Lower tumor rupture risk.

Disadvantages:

Greater trauma, longer recovery, higher complication rates (e.g., infection, bowel obstruction).

Increased need for postoperative pain management.

④Key Evidence and Guidelines

Evidence supporting MIS:

Comparable survival rates to laparotomy in early-stage disease (LANCE trial).

Robotic assistance improves precision in complex maneuvers (e.g., lymphadenectomy).

Evidence supporting laparotomy:

Higher R0 rates in advanced disease (LION trial).

Potential increased recurrence risk with MIS in high-grade cancers (some retrospective studies).

Guidelines:

NCCN: MIS cautiously allowed for early-stage disease by experienced teams; laparotomy preferred for advanced cases.

ESMO: MIS restricted to rigorously selected Stage I patients with intraoperative frozen section confirmation.

⑤Decision-Making Factors

Multidisciplinary evaluation: Integrate imaging, pathology, and genetic data.

Intraoperative flexibility: Convert to laparotomy if metastasis is detected during MIS.

Informed consent: Disclose risks of recurrence, staging accuracy, and fertility implications.

⑥Special Populations

Pregnancy-associated ovarian cancer: Laparoscopy may be considered in the second trimester.

Elderly/frail patients: Balance surgical benefits with tolerance; MIS may be preferable.

Medical:

11.Which patients with ovarian cancer should receive neoadjuvant chemotherapy?

①Indications

Advanced disease (FIGO III/IV):

Imaging (CT/PET-CT) or laparoscopy suggests suboptimal cytoreduction (residual disease >1 cm or low likelihood of R0).

Extensive peritoneal carcinomatosis, omental caking, mesenteric root involvement, or liver parenchymal metastases.

High surgical risk:

Severe cardiopulmonary comorbidities, malnutrition, or poor performance status (ECOG ⩾2).

Rapid progression/severe symptoms:

Massive ascites, bowel obstruction, or systemic symptoms (e.g., cachexia) requiring rapid palliation.

②Contraindications

Allergic to platinum-based or taxane drugs, or with severe organ dysfunction (such as renal failure, bone marrow suppression).

Having contraindications for chemotherapy (such as uncontrolled active infection).

③Preoperative Evaluation

Imaging: Contrast CT or PET-CT to assess disease burden; diagnostic laparoscopy for resectability.

Pathologic confirmation: Ascites cytology or biopsy to confirm high-grade serous carcinoma.

Multidisciplinary team (MDT) review: Collaborative decision-making with gynecologic oncologists, radiologists, and medical oncologists.

④Treatment Protocol

Chemotherapy regimen: Platinum-taxane combination (e.g., carboplatin + paclitaxel) every 3 weeks for 3-4 cycles.

Response assessment:

CT imaging to evaluate tumor shrinkage.

≥50% decline in CA-125 indicates response.

Surgical timing: Interval debulking surgery (IDS) 3-4 weeks after last NACT cycle.

⑤Prognosis and Evidence

Survival outcomes:

CHORUS and EORTC 55971 trials showed similar OS between NACT+IDS and primary surgery, but lower surgical morbidity with NACT.

NACT improves resectability in patients unlikely to achieve R0 upfront.

Risks:

Chemoresistance may lead to progression.

Delayed surgery if NACT fails.

⑥Patient Counseling

Clarify goals of NACT (tumor reduction to optimize surgery).

Discuss potential side effects (myelosuppression, neuropathy, hypersensitivity).

Emphasize the need for postoperative adjuvant chemotherapy (total 6 cycles).

12.When should HIPEC be used in ovarian cancer?

①Indications

Primary treatment:

Advanced disease (FIGO III): Combined with HIPEC after optimal primary cytoreduction (residual ≤1 cm or R0).

Interval debulking surgery (IDS) post-neoadjuvant chemotherapy: Especially for stage III patients with good chemoresponse and R0/R1 resection.

Recurrent disease:

Platinum-sensitive recurrence: Consider HIPEC after secondary optimal cytoreduction (limited evidence, case-dependent).

High-risk histologies:

High-grade serous carcinoma or primary peritoneal cancer with peritoneal spread.

②Contraindications

Extensive extraperitoneal metastases (e.g., liver/lung metastases).

Poor surgical tolerance: Severe cardiopulmonary disease, coagulopathy, or ECOG ≥2.

Bowel obstruction/severe adhesions: Risk of uneven drug distribution or complications.

Renal impairment: Contraindicated for nephrotoxic agents (e.g., cisplatin).

③Protocol and Parameters

Drugs: Cisplatin (75-100 mg/m^2) ± paclitaxel, heated to 41-43°C.

Duration: 60-90 minutes intraoperatively or post-resection.

Technical notes:

Ensure full peritoneal coverage (open/closed technique).

Monitor temperature, hemodynamics, and electrolytes continuously.

④Evidence

OVHIPEC-1 trial: Stage III patients showed 12-month OS improvement with HIPEC (45.7 vs. 33.7 months).

KELIM score guidance: Greater benefit if KELIM ≥0.5 (chemosensitive) post-neoadjuvant therapy.

Limitations: No proven survival benefit in stage IV or platinum-resistant disease.

⑤Prognosis and Risks

Survival benefit: 15%-20% higher 5-year OS in R0 patients.

Complications:

Short-term: Ileus, renal injury, myelosuppression.

Long-term: Adhesions, chronic abdominal pain.

Recurrence patterns: Reduces peritoneal recurrence but not distant metastases.

⑥Patient Counseling

Discuss survival benefits vs. surgical risks (e.g., prolonged anesthesia).

Stress the need for adjuvant chemotherapy (if incomplete cycles).

Explore alternatives (e.g., IV chemotherapy + targeted therapy).

13.Which patients with ovarian cancer should receive bevacizumab for maintenance therapy?

①Indications

Newly diagnosed advanced disease (FIGO III/IV):

Suboptimal cytoreduction (residual disease >1 cm) after PDS or IDS.

High-grade serous, endometrioid, or primary peritoneal carcinoma.

Combined with chemotherapy followed by maintenance (per GOG-0218 and ICON7 trials).

Platinum-sensitive recurrent disease:

Effective response to platinum-based chemotherapy (CR/PR), especially if bevacizumab-naïve.

High-risk features:

Patients with ascites or extensive peritoneal spread (anti-angiogenic benefit).

②Contraindications

Cardiovascular disease: Uncontrolled hypertension (SBP >150 mmHg), recent arterial thromboembolism (MI, stroke).

Bowel perforation risk: Active bowel obstruction, poor anastomotic healing, or extensive bowel resection.

Renal impairment: Proteinuria ⩾2 g/24h or nephrotic syndrome.

Bleeding risk: Recent major surgery with unhealed wounds, active GI bleeding.

Pregnancy/lactation: Potential fetal harm.

③Treatment Protocol

Chemotherapy phase:

Dose: 15 mg/kg every 3 weeks (with carboplatin/paclitaxel).

Maintenance phase:

Dose: 15 mg/kg (frontline) or 7.5-15 mg/kg (recurrent), continued until progression or toxicity.

Duration: Typically 15-22 months (adjusted for tolerance).

④Evidence

GOG-0218 trial: Bevacizumab maintenance improved PFS in advanced disease (14.1 vs. 10.3 months).

ICON7 trial: OS benefit in high-risk subgroups (stage IV or suboptimally debulked III; median OS +4.8 months).

Platinum-sensitive recurrence (OCEANS trial): Maintenance extended PFS (12.4 vs. 8.4 months).

⑤Prognostic Factors

Biomarkers:

Greater benefit in BRCA wild-type or HRD-negative tumors.

High VEGF expression correlates with response.

Survival outcomes:

Median PFS gain 3-6 months, limited OS improvement (significant only in select subgroups).

⑥Patient Counseling

Toxicity management: Monitor hypertension, proteinuria, bleeding/thrombosis.

Survival trade-offs: Emphasize PFS vs. OS benefits and quality-of-life impact.

Alternatives: PARP inhibitors (e.g., olaparib) preferred for HRD-positive patients.

14.Should patients with recurrent epithelial ovarian cancer who had received one prior PARP inhibitor as maintenance therapy be retreated with a PARP inhibitor?

For patients with recurrent epithelial ovarian cancer who have previously received a PARP inhibitor as maintenance therapy, whether to use a PARP inhibitor again should be considered based on the following factors:

①Clinical Evidence

OReO Trial: This phase IIIb study showed that re-administering olaparib as maintenance therapy in patients who had previously received a PARP inhibitor for maintenance therapy can extend progression-free survival (PFS), particularly in patients with BRCA mutations (median PFS 4.3 months vs. placebo 2.8 months). However, overall survival (OS) did not improve significantly, and the benefit was limited.

Cross-resistance: There may be cross-resistance between different PARP inhibitors. Data on switching to another PARP inhibitor is limited, and current evidence mainly supports rechallenge with the same drug.

②Molecular Characteristics

BRCA Mutation/HRD Status: Patients with BRCA mutations or HRD-positive status may be more likely to benefit from retreatment. It is essential to confirm whether the molecular status has changed after recurrence (e.g., secondary mutations or loss of HRD status).

Platinum Sensitivity: PARP inhibitors are typically used for maintenance therapy in platinum-sensitive relapsed patients (those with a remission duration ⩾6 months after chemotherapy). If the patient remains platinum-sensitive, there may be a higher potential for retreatment success.

③Treatment History and Toxicity

Response to Previous Treatment: If the patient had a prolonged remission and good tolerance to the initial PARP inhibitor, they may be more suitable for rechallenge.

Toxicity Management: The risk of cumulative toxicity (e.g., bone marrow suppression) should be assessed, especially after prolonged use.

④Guidelines and Clinical Practice

NCCN/ESMO Guidelines: Current guidelines do not explicitly recommend routine

rechallenge but indicate that it may be considered on a case-by-case basis in specific circumstances (e.g., platinum-sensitive relapse, molecular characteristics supporting retreatment).

Clinical Trial Participation: Patients are encouraged to participate in clinical trials exploring PARP inhibitor retreatment or combination therapies.

Conclusion

PARP inhibitor retreatment may be cautiously considered under the following conditions:

Platinum-sensitive relapse with sustained response to prior PARP inhibitor therapy (e.g., >6 months);

Persistent BRCA mutation or HRD-positive status;

Good tolerance, with no significant cumulative toxicity;

Individualized decision-making after multidisciplinary discussion, with clear communication of the limitations of the available evidence.

Ultimately, the decision should be based on the patient's specific situation, molecular characteristics, and the latest research developments. Participation in clinical trials to explore better strategies is highly recommended.

15.Should patients with ovarian cancer be treated with immunotherapy or not?

①Indications

Biomarker-positive patients:

MSI-H/dMMR: Pembrolizumab is preferred (per KEYNOTE-158 trial).

TMB-H (⩾10 mut/Mb): Potential benefit from nivolumab.

Platinum-resistant recurrence:

Combination regimens (e.g., immunotherapy + chemotherapy/anti-angiogenics/PARP inhibitors) may extend PFS (e.g., JAVELIN Ovarian 200 trial).

Clinical trial candidates:

Encourage enrollment in trials exploring novel immunotherapies (e.g., bispecific antibodies, CAR-T).

②Contraindications

Active autoimmune diseases: SLE, rheumatoid arthritis (risk of exacerbation).

Severe immune-related adverse event (irAE) history: e.g., pneumonitis, colitis.

Organ dysfunction: Uncontrolled hepatic/renal failure or myelosuppression (ANC $<1.5\times10^9$ /L).

Steroid dependency: Chronic high-dose corticosteroids (>10 mg/day prednisone equivalent).

③Treatment Protocols

Monotherapy:

Pembrolizumab (200 mg IV q3w) for MSI-H/dMMR.

Nivolumab (240 mg IV q2w) for PD-L1+ or TMB-H.

Combination therapy:

Immunotherapy + chemotherapy (e.g., paclitaxel + carboplatin + atezolizumab).

Immunotherapy + anti-angiogenics (e.g., bevacizumab + atezolizumab).

Immunotherapy + PARP inhibitors (e.g., durvalumab + olaparib).

④Evidence

KEYNOTE-100 trial: Pembrolizumab showed 8% ORR in recurrent ovarian cancer (14% in PD-L1+ subgroup).

NRG-GY003 trial: Nivolumab monotherapy achieved 15% ORR in platinum-resistant cases (median PFS 3.5 months).

IMagyn050 trial: Atezolizumab + bevacizumab + chemotherapy did not significantly improve PFS in frontline advanced disease.

Limitations: Low overall response rates (<20%), lack of OS benefit.

⑤Prognostic Factors

Biomarkers:

MSI-H/dMMR tumors have 30%-50% ORR.

PD-L1 expression (CPS ≥1) may predict partial response.

Tumor microenvironment:

CD8+ T-cell infiltration and high TILs correlate with better outcomes.

⑥Patient Counseling

Efficacy expectations: Emphasize biomarker-driven selection and limited overall benefit.

Toxicity management: Early recognition of irAEs (rash, thyroid dysfunction, hepatitis).

Cost/access: Some agents lack ovarian cancer approval; consider financial/insurance barriers.

Alternatives: Targeted therapy (e.g., mirvetuximab) or clinical trials for platinum-resistant cases.

Surveillance:

16.How should patients with ovarian cancer be followed after treatment is complete?

①Follow-Up Objectives

Detect recurrence: Early identification of asymptomatic relapse for timely intervention.

Manage treatment-related complications: Address chemotherapy-induced neuropathy, myelosuppression, or surgical sequelae.

Improve quality of life: Focus on psychological health, nutrition, bone health, and sexual function.

②Follow-Up Schedule and Components

First 2 years:

Every 3-4 months:

History and physical exam: Assess symptoms (bloating, pain, weight loss), pelvic examination.

Tumor markers: CA-125 (if elevated pretreatment), HE4 (optional).

Imaging: Ordered only for symptoms, rising CA-125, or clinical suspicion (e.g., contrast CT/MRI).

Years 3-5:

Every 4-6 months: Same as above, with reduced imaging frequency.

Beyond 5 years:

Annually: Focus on long-term complications (cardiovascular disease, osteoporosis, secondary cancers).

③Recurrence Monitoring

Symptom alerts: New/worsening ascites, bowel obstruction, pelvic mass, dyspnea (pleural effusion), or systemic decline.

CA-125 trends:

Persistent elevation (⩾2x ULN) warrants evaluation, excluding non-malignant causes.

Asymptomatic slow rise may be observed without immediate intervention.

Imaging confirmation:

CT/MRI findings require PET-CT for systemic staging.

Biopsy suspicious lesions for pathology and molecular profiling (e.g., BRCA, HRD).

④Long-Term Complication Management

Menopausal symptoms:

Hormone replacement therapy (HRT) individualized (caution in BRCA carriers).

Non-hormonal options (e.g., SSRIs) for vasomotor symptoms.

Bone health:

Annual DEXA scans, calcium (1200 mg/day), and vitamin D (800 IU/day).

Bisphosphonates (e.g., zoledronate) or denosumab for osteoporosis prevention.

Cardiovascular risk:

Monitor blood pressure, lipids, glucose; promote smoking cessation, exercise, and Mediterranean diet.

⑤Psychosocial Support and Quality of Life

Mental health screening: Refer to psychologists/support groups for anxiety/depression.

Nutrition counseling: Avoid obesity (BMI >30 increases recurrence risk); emphasize plant-based protein and fiber.

Sexual health:

Topical estrogen for vaginal dryness; lubricants for dyspareunia.

Fertility counseling if applicable.

⑥Genetic Counseling and Family Screening

Genetic testing: Recommend BRCA1/2, HRD, and other ovarian cancer genes (e.g., RAD51C/D) if not previously done.

Family risk assessment: First-degree relatives require genetic counseling and screening (e.g., TVUS + CA-125).

⑦Patient Education

Symptom diary: Document bloating, urinary frequency, fatigue, and report promptly.

Lifestyle modifications: Avoid smoking, limit alcohol, maintain moderate exercise (150 minutes/week).

Follow-up adherence: Stress the importance of regular visits for early detection.

17.How to define platinum-resistant recurrent ovarian cancer?

①Core Definition

Platinum-Resistant: Disease progression or recurrence occurs within 6 months after the last dose of platinum-based chemotherapy (e.g., carboplatin, cisplatin).

Platinum-Refractory: Progression during or within 4 weeks of completing initial platinum therapy, indicating no response.

②Diagnostic Criteria

Imaging Evidence:

CT, MRI, or PET-CT showing ⩾20% increase in target lesions (per RECIST 1.1) or new lesions.

Tumor Markers:

Persistent CA-125 elevation (⩾2x upper limit of normal), excluding non-malignant causes (e.g., infection).

Pathologic Confirmation:

Biopsy of recurrent lesions (if indicated) to assess histology and molecular markers (e.g., BRCA1/2, HRD status).

③Clinical Features

Typical Presentation:

Rapidly progressing ascites, bowel obstruction, extensive peritoneal carcinomatosis, or liver metastases.

Low response rates to subsequent platinum rechallenge (＜10%).

Molecular Profile:

More common in BRCA wild-type or HRD-negative tumors.

Potential mechanisms include clonal evolution or acquired resistance (e.g., drug efflux pump activation).

④Distinction from Other Recurrent Types

Platinum-Sensitive: Platinum-free interval (PFI) ⩾6 months, likely responsive to platinum retreatment.

Partially Platinum-Sensitive: PFI 6-12 months, intermediate efficacy.

⑤Prognostic Factors

PFI Duration: Shorter PFI (e.g., <3 months) correlates with worse outcomes (median OS ~9-12 months).

Tumor Burden: Liver/lung metastases or extensive peritoneal disease reduces survival.

Molecular Subtypes:

BRCA1/2 mutations or HRD positivity may retain partial response to PARP inhibitors or combination therapies.

⑥Treatment Challenges & Strategies

Chemotherapy Options:

Non-platinum monotherapy (e.g., gemcitabine, docetaxel) has low response rates (10%-15%).

Pegylated liposomal doxorubicin + bevacizumab may extend PFS.

Targeted Therapies:

Bevacizumab + chemotherapy improves ORR.

PARP inhibitors (e.g., olaparib) show limited efficacy unless BRCA-mutated.

Immunotherapy:

Immune checkpoint inhibitors (e.g., pembrolizumab) for MSI-H/dMMR patients.

Combination trials (e.g., immunotherapy + anti-angiogenics) ongoing (e.g., NRG-GY009).

⑦Patient Management

Multidisciplinary Evaluation: Collaborative planning with radiologists, pathologists, and oncologists.

Supportive Care: Paracentesis, nutritional support, pain control, and psychological counseling.

Clinical Trial Priority: Encourage enrollment in trials exploring novel agents (e.g., antibody-drug conjugates, bispecific antibodies).

18.What is the management strategy for biochemical relapse of ovarian cancer?

①Definition and Diagnostic Criteria

Biochemical relapse: Persistent elevation of CA-125 (⩾2x ULN) without radiologic evidence of disease.

Diagnostic prerequisites: Exclude non-malignant causes (e.g., infection, liver dysfunction).

②Evaluation Steps

Serial monitoring: Repeat CA-125 every 2-4 weeks to confirm rising trend.

Imaging reassessment: Perform contrast CT or PET-CT if CA-125 continues to rise.

Molecular profiling: Test BRCA1/2, HRD, and PD-L1 to guide targeted therapy.

③Treatment Decision Factors

Patient factors:

Symptoms (ascites, pain): Immediate intervention if symptomatic.

Platinum-free interval (PFI): ⩾6 months (platinum-sensitive) vs. <6 months (platinum-resistant).

CA-125 kinetics: Rapid doubling (e.g., monthly) indicates aggressive relapse.

④Management Strategies

Delayed treatment (watchful waiting):

Candidates: Asymptomatic patients with slow CA-125 rise and PFI ⩾6 months.

Evidence: MRC OV05/EORTC 55955 trials showed no OS detriment with delayed treatment.

Immediate treatment:

Chemotherapy:

Platinum-sensitive: Carboplatin + paclitaxel (or pegylated liposomal doxorubicin).

Platinum-resistant: Gemcitabine + bevacizumab or non-platinum monotherapy.

Targeted therapy:

PARP inhibitors (olaparib, niraparib) for BRCA-mutated or HRD-positive cases.

Bevacizumab + chemotherapy to enhance response rates.

Clinical trials: Enroll in studies exploring novel agents (e.g., antibody-drug conjugates, dual immunotherapy).

⑤Follow-Up and Monitoring

Imaging frequency: Contrast CT every 3 months until lesions are identified or CA-125 stabilizes.

Quality of life: Address treatment toxicities (neuropathy, fatigue) and psychological well-being.

⑥Patient Counseling

Risk-benefit analysis: Explain trade-offs between delaying treatment (prolonging symptom-free periods) and immediate intervention (potential survival benefit with side effects).

Molecular testing: Highlight the role of BRCA/HRD status in PARP inhibitor efficacy.

Psychological support: Provide counseling to manage anxiety during surveillance.

19.What are the independent prognostic factors for ovarian cancer?

①Clinicopathological Factors

FIGO Stage:

Stage I: 5-year survival >90%; Stage IV: <20%.

Advanced stage correlates with worse outcomes (peritoneal spread, distant metastasis).

Residual Disease After Surgery:

Median OS: ~60 months for R0 vs. <24 months for R2 (>1 cm residual).

Histology and Grade:

High-grade serous carcinoma has poorer prognosis; low-grade serous, mucinous, and germ cell tumors fare better.

High-grade (G3) tumors are more aggressive than G1-2.

Age:

Patients <65 years have better survival than ⩾65 years (due to treatment tolerance/comorbidities).

②Molecular Biomarkers

BRCA1/2 Mutations:

BRCA1/2 carriers have longer OS (enhanced PARP inhibitor sensitivity).

BRCA2 may confer better prognosis than BRCA1.

Homologous Recombination Deficiency (HRD):

HRD positivity predicts better response to platinum and PARP inhibitors (prolonged PFS).

Other Genetic Alterations:

TP53 mutations (common in high-grade tumors) indicate poor prognosis.

CCNE1 amplification correlates with platinum resistance and shorter survival.

③Treatment Response Factors

Platinum Sensitivity:

Platinum-sensitive recurrence (PFI ⩾6 months): Median OS 24-36 months vs. 9-12 months for platinum-resistant.

CA-125 Kinetics:

Post-chemotherapy CA-125 normalization (≤35 U/mL) predicts superior survival.

CA-125 half-life <20 days suggests favorable treatment response.

④Tumor Microenvironment and Immune Features

Tumor-Infiltrating Lymphocytes (TILs):

High CD8+ T-cell infiltration correlates with longer OS (notably in high-grade serous carcinoma).

PD-L1 Expression:

PD-L1 positivity (CPS ≥1) may predict immunotherapy response, but prognostic value remains debated.

⑤Patient Status and Comorbidities

Performance Status (ECOG):

ECOG 0-1 patients have significantly better survival than ECOG ≥2.

Ascites and Bowel Obstruction:

Massive ascites or recurrent bowel obstruction indicates aggressive disease.

Nutritional Status:

Hypoalbuminemia (<3.5 g/dL) or BMI <18.5 reduces survival rates.

⑥Treatment Modalities

Surgical Completeness:

Optimal debulking (residual ≤1 cm) by gynecologic oncologists improves outcomes.

Chemotherapy Intensity:

Platinum-based combination therapy (carboplatin + paclitaxel) extends survival vs. monotherapy.

Maintenance Therapy:

PARP inhibitors (olaparib, niraparib) or bevacizumab maintenance prolongs PFS.

20.How to manage chemotherapy-associated adverse events in patients with ovarian cancer?

①Hematologic Toxicity

Neutropenia:

Prophylaxis: Use G-CSF (e.g., filgrastim) for high-risk patients.

Treatment: Febrile neutropenia requires hospitalization and broad-spectrum antibiotics (e.g., piperacillin/tazobactam).

Anemia:

Mild: Oral iron or erythropoiesis-stimulating agents (ESAs).

Severe (Hb <8 g/dL): Red blood cell transfusion with iron status evaluation.

Thrombocytopenia:

Prophylaxis: Platelet transfusion if bleeding risk is high.

Treatment: Transfuse for platelets $<10\times10^9/L$; TPO agonists (e.g., romiplostim) for chronic cases.

②Gastrointestinal Effects

Nausea/Vomiting:

Acute phase: Combine 5-HT3 antagonist (e.g., ondansetron) + NK1 antagonist (e.g., aprepitant) + dexamethasone.

Delayed phase (24-120 hrs): Continue dexamethasone or use olanzapine.

Diarrhea:

Mild: Loperamide (4 mg initially, then 2 mg every 2 hrs, max 16 mg/day).

Severe (≥4 episodes/day): Hold chemotherapy, hydrate IV, and administer octreotide (100 μg SC tid).

③Neurotoxicity

Peripheral Neuropathy:

Prevention: Vitamin B1/B6/B12 supplementation; avoid cold exposure (taxane-related).

Treatment: Dose reduction (e.g., paclitaxel); gabapentin/pregabalin for pain.

Oxaliplatin-Induced Neuropathy:

Acute: Avoid cold exposure; calcium/magnesium infusions may help.

Chronic: Extend intervals or switch to carboplatin.

④Hypersensitivity Reactions

Taxane Premedication: Dexamethasone (20 mg IV), diphenhydramine (50 mg), H2 blocker (e.g., famotidine).

Acute Reaction: Stop infusion, administer epinephrine (0.3-0.5 mg IM), and methylprednisolone (125 mg IV).

⑤Nephrotoxicity (Cisplatin)

Prevention: Hydration (⩾3 L/day), monitor urine output, magnesium supplementation.

Treatment: Avoid nephrotoxic drugs (e.g., NSAIDs); consider carboplatin substitution.

⑥Alopecia

Prevention: Scalp cooling (partial efficacy with anthracyclines/taxanes).

Support: Wigs, psychological counseling.

⑦Other Specific Toxicities

Cardiotoxicity (Anthracyclines): Monitor LVEF; use dexrazoxane for cardioprotection.

Hand-Foot Syndrome (Liposomal Doxorubicin): Urea-based creams; dose reduction if severe.

⑧Supportive Care

Nutrition: High-protein diet, oral supplements (e.g., Ensure).

Psychological Support: Screen for anxiety/depression; refer to specialists if needed.
