# Supplementary material for: Decoding AI Competence: Benchmarking Large Language Models (LLMs) in Ovarian Cancer Diagnosis and Treatment—A Systematic Evaluation of Generative AI Accuracy and Completeness
Source: Diagnostics (Basel). 2026 Feb 20;16(4):616. doi: 10.3390/diagnostics16040616 (PMC12939671; doi:10.3390/diagnostics16040616)
Supplement: Supplementary file 1 [file diagnostics-16-00616-s001.zip › FileS2-Answers from Doubao.pdf]

Risk Factors and Prevention:

1.What are the risk factors for ovarian cancer?

Genetic factors: Approximately 10% of ovarian cancers are related to genetics. Women carrying BRCA1 or BRCA2 gene mutations have a significantly increased risk of ovarian cancer. In addition, hereditary diseases such as Lynch syndrome can also increase the risk of ovarian cancer.

Age factors: The incidence of ovarian cancer increases with age, mostly occurring in women over 50 years old, and the peak incidence is between 60 and 79 years old.

Reproductive history: Women who have never given birth have a relatively higher risk of ovarian cancer. Multiple pregnancies and breastfeeding may reduce the risk of ovarian cancer, because the ovaries ovulate less during pregnancy and breastfeeding, which can reduce the damage and repair of ovarian epithelial cells, thereby reducing the risk of cancer.

Hormonal factors: Long-term use of estrogen replacement therapy may increase the risk of ovarian cancer, especially when progesterone is not used at the same time. Patients with polycystic ovary syndrome may also have an increased risk of ovarian cancer due to hormonal imbalance.

Lifestyle: Unhealthy lifestyles such as high-fat diet, obesity and lack of exercise may be related to the occurrence of ovarian cancer. Long-term smoking can damage ovarian tissue and increase the risk of cancer. Excessive alcohol consumption may also have an adverse effect on ovarian health.

Environmental factors: Long-term exposure to environmental carcinogens such as asbestos and talcum powder may increase the risk of ovarian cancer.

History of other diseases: Women with a history of breast cancer, endometrial cancer and other gynecological cancers may have an increased risk of ovarian cancer. Chronic pelvic inflammation that persists for a long time may lead to repeated inflammatory stimulation of ovarian tissue, increasing the risk of cancer.

2.What genetic and molecular testing is recommended in patients with ovarian cancer?

BRCA1 and BRCA2 gene testing: BRCA1 and BRCA2 are important tumor suppressor genes, and their mutations are closely related to the occurrence of ovarian cancer. Testing these two genes helps to assess the patient's genetic risk and can also provide a basis for subsequent targeted therapies, such as the use of PARP inhibitors.

Lynch syndrome-related gene testing: This includes genes such as MLH1, MSH2, MSH6 and PMS2. Patients with Lynch syndrome have an increased risk of ovarian cancer and other malignancies. Detecting these genes helps to identify potential genetic causes and guide cancer prevention in family members.

Homologous recombination repair deficiency (HRD) testing: In addition to BRCA1/2 gene mutations, other genetic alterations that lead to homologous recombination repair deficiency can also affect the treatment and prognosis of ovarian cancer. HRD testing can assess the homologous recombination repair status of tumor cells and help predict the efficacy of drugs such as PARP inhibitors.

Gene expression profiling: By analyzing the gene expression in tumor cells, ovarian cancer can be divided into different molecular subtypes, such as serous, endometrioid, clear cell carcinoma, etc., which helps to more accurately judge the prognosis and formulate treatment strategies.

Copy number variation testing: Detecting changes in the copy number of genes in tumor cells, such as the amplification of certain oncogenes or the deletion of tumor suppressor genes, may provide new targets and ideas for treatment.

Microsatellite instability (MSI) testing: High MSI expression may indicate a better response to immunotherapy such as immune checkpoint inhibitors and also has certain predictive value in ovarian cancer.

3.How much do BRCA mutations increase risk for ovarian cancer?

BRCA1 gene mutation: For women carrying the BRCA1 gene mutation, the lifetime risk of developing ovarian cancer is approximately 40% - 60%. That is to say, if there are 100 women carrying the BRCA1 gene mutation, then about 40 to 60 of them may develop ovarian cancer in their lifetime.

BRCA2 gene mutation: For women carrying the BRCA2 gene mutation, the lifetime risk of developing ovarian cancer is approximately 15% - 30%. That is, among every 100 women carrying the BRCA2 gene mutation, about 15 to 30 of them will develop ovarian cancer in their lifetime.

4.Who should be tested for BRCA mutations?

People with a family history of ovarian cancer: If there are ovarian cancer patients in the family, especially if first-degree relatives (such as mothers, sisters, daughters) have ovarian cancer, then other family members should consider undergoing BRCA gene mutation testing, as there may be an increased risk of disease due to genetic factors.

People with a family history of breast cancer: If there are multiple members with breast cancer in the family, especially if the age of onset is early (such as under 50 years old), or if there are male breast cancer patients, it is necessary for their relatives to undergo BRCA gene mutation testing, because BRCA gene mutations are closely related to the occurrence of both breast cancer and ovarian cancer.

Patients with ovarian cancer: For patients who have been diagnosed with ovarian cancer, testing for BRCA gene mutations helps to understand the pathogenesis of the tumor, guide the selection of subsequent treatment plans, such as whether they are suitable for targeted drugs such as PARP inhibitors, and also assess the disease risk of family members.

Patients with breast cancer: Especially patients with triple-negative breast cancer or those with an early age of onset, undergoing BRCA gene mutation testing can help determine whether there is a genetic cause of the disease, which is of great significance for subsequent treatment and family genetic risk assessment.

People with a family history of other related hereditary cancer syndromes: If there are patients with hereditary cancer syndromes related to BRCA gene mutations in the family, such as Lynch syndrome and Li - Fraumeni syndrome, other family members should also consider undergoing testing.

5.How can you prevent cancer if you test positive for BRCA mutations?

①Strengthen monitoring

Regular gynecological examinations: This includes pelvic examinations, transvaginal ultrasound examinations and serum CA125 tests to detect early ovarian cancer and other related lesions. It is generally recommended to start from the age of 30 to 35, and have an examination every six months or a year.

Breast screening: For women, it is recommended to start breast screening from the age of 25 to 30, including annual breast magnetic resonance imaging (MRI), mammography and breast ultrasound examinations. If necessary, clinical breast examinations and breast self-examinations are also needed.

②Preventive surgery

Prophylactic bilateral salpingo-oophorectomy: For BRCA gene mutation carriers who have completed childbearing or do not plan to have children, after fully weighing the pros and cons, prophylactic bilateral salpingo - oophorectomy can be considered at the age of 35 to 40 or 5 to 10 years earlier than the earliest age of onset in the family. This can significantly reduce the risk of ovarian and fallopian tube cancer and also has a certain effect in reducing the risk of breast cancer.

Prophylactic mastectomy: For high-risk women with BRCA1/2 gene mutations, prophylactic mastectomy can be considered after fully understanding the pros and cons of the surgery and psychological evaluation. However, this surgery needs to strictly control the indications and contraindications.

③Lifestyle adjustments

Healthy diet: Increase the intake of vegetables, fruits, whole grains and high-fiber foods, and reduce the intake of high-fat, high-sugar and processed meats, which helps to maintain good health and reduce the risk of cancer.

Appropriate exercise: Perform at least 150 minutes of moderate-intensity aerobic exercise per week, such as brisk walking, jogging, swimming, etc. Strength training can also be appropriately combined, which helps to improve the body's immunity and reduce the risk of cancer.

Quit smoking and limit alcohol: Smoking and excessive alcohol consumption are risk factors for many cancers. Quitting smoking and avoiding excessive alcohol consumption can reduce damage to the body and reduce the risk of cancer.

Weight control: Maintain a healthy body mass index (BMI) and avoid obesity, as obesity is associated with an increased risk of many cancers.

④Drug prevention

Chemopreventive drugs: Under the guidance of a doctor, some people can consider using some chemopreventive drugs, such as tamoxifen, raloxifene, etc., which have a certain effect in reducing the risk of breast cancer. However, these drugs have strict indications and adverse reactions and need to be used with caution.

Surgical:

6.What are the principles of surgery for newly diagnosed invasive epithelial ovarian cancer involving the pelvis and upper abdomen (stage ≥IIB)?

①Principles of cytoreductive surgery

Complete tumor resection: Resect all visible tumor lesions as much as possible, including the primary tumor and metastatic foci. For tumors in the pelvis, it is necessary to remove the uterus, bilateral adnexa, greater omentum, appendix and the involved parts of the pelvic organs such as the bladder and rectum that may be invaded. In the upper abdomen, metastatic foci on the surface of the diaphragm, liver surface, spleen and other parts should be resected, and retroperitoneal lymph nodes should be dissected, including pelvic lymph nodes and para-aortic lymph nodes.

Achieve satisfactory cytoreduction effect: Ideally, the diameter of the remaining tumor lesions should be less than 1 cm, and it is best to have no visible residual lesions, so as to improve the patient's survival rate and prognosis. If satisfactory cytoreduction cannot be achieved in the initial surgery, neoadjuvant chemotherapy can be considered, and then the surgery can be performed after the tumor shrinks.

②Principles of protecting important organ functions

Preserve normal tissues as much as possible: During the process of tumor resection, the functions of surrounding normal tissues and organs should be protected as much as possible. For example, for young patients with fertility needs, the contralateral ovary and uterus can be considered for preservation to retain fertility function if the condition permits after evaluation. For important organs such as the bladder and rectum, if the tumor invasion is superficial, try to use the method of peeling or partial resection to avoid total organ resection and maintain their normal urination and defecation functions.

Fine operation to reduce damage: The surgical operation should be fine, and advanced surgical techniques and equipment such as laparoscopy or robotic-assisted surgery can be used, which is helpful for more accurate tissue separation, reducing the damage to surrounding blood vessels, nerves and other structures, reducing the risk of surgical complications and improving the patient's quality of life after surgery.

③Principles of staging surgery

Comprehensive and accurate staging: Staging the tumor comprehensively through surgery to clarify the extent of tumor involvement and metastasis, which is crucial for formulating subsequent treatment plans and evaluating prognosis. In addition to the above tumor resection and lymph node dissection, biopsies of multiple sites in the abdominal cavity, such as the peritoneum of the pelvis and abdomen, under the diaphragm, and the porta hepatis, are also required to determine whether there are micrometastases.

Follow the standardized staging process: The surgical process should strictly follow the staging criteria and surgical operation specifications of the International Federation of Gynecology and Obstetrics (FIGO) to ensure the accuracy and consistency of staging, which is convenient for the comparison of treatment effects and academic exchanges among different medical institutions.

④Principles of multidisciplinary collaboration

Combine multidisciplinary teams: Due to the complexity of such ovarian cancer, surgery often requires the collaboration of multidisciplinary teams such as obstetrics and gynecology, general surgery, urology, and hepatobiliary surgery. Experts from each discipline, based on their professional knowledge and experience, jointly formulate the surgical plan, perform their respective duties during the surgery, and complete the surgery together to improve the success rate and safety of the surgery.

Comprehensive treatment planning: It is also necessary to cooperate with departments such as medical oncology and radiotherapy before and after surgery to formulate a comprehensive treatment plan. Evaluate whether neoadjuvant chemotherapy is needed before surgery, and decide whether adjuvant chemotherapy, targeted therapy or radiotherapy is needed after surgery according to the pathological results and the patient's physical condition, so as to improve the patient's treatment effect and survival rate.

7.What is the surgical protocol for risk-reducing salpingo-oophorectomy?

①Preoperative assessment

Risk assessment: Conduct a comprehensive risk assessment of the patient, including family medical history, genetic test results (such as BRCA1/2 gene mutations), age, reproductive history, past medical history, etc., to determine the necessity and risk level of salpingo - oophorectomy for the patient.

Imaging examination: Perform imaging examinations such as pelvic ultrasound, MRI or CT to understand the shape, size of the bilateral fallopian tubes and ovaries, and whether there are abnormal masses, etc., to provide reference for the surgery.

Laboratory tests: Conduct blood routine, coagulation function, liver and kidney function, tumor markers (such as CA125, HE4, etc.) and other tests to evaluate the patient's physical condition and rule out other possible diseases.

Fertility status assessment: For patients with fertility needs, it is necessary to comprehensively evaluate their fertility ability and fertility intention, inform them of the impact of the surgery on fertility, and help patients make decisions.

②Selection of surgical timing

Premenopausal women: It is generally recommended to perform the surgery after childbearing and before menopause, which can minimize the risk of ovarian and fallopian tube cancer. For high-risk individuals carrying BRCA gene mutations, surgery can be considered after the age of 35 - 40 or 5 - 10 years earlier than the age of onset in the family.

Postmenopausal women: The timing of surgery is relatively more flexible, but factors such as the patient's overall health status and surgical risk also need to be considered comprehensively. Generally, the surgery can be arranged as soon as possible after the discovery of high-risk factors if there are no special circumstances.

③Surgical methods

Laparoscopic surgery: It is a commonly used surgical method at present, with the advantages of less trauma, quick recovery and short hospital stay. Several small incisions are made in the abdomen, and a laparoscope and surgical instruments are inserted to perform the resection of the fallopian tubes and ovaries.

Open surgery: It is suitable for some patients with complex conditions, extensive adhesions or other surgical contraindications who are not suitable for laparoscopic surgery. The operation is carried out through a midline or transverse incision in the lower abdomen to enter the abdominal cavity. It can better expose the surgical field and is convenient for dealing with complex situations.

Robot-assisted surgery: The operation is carried out using a robotic surgical system, which has the advantages of more precise and flexible operation, but the cost is relatively high and the technical requirements for the surgical team are high.

④Surgical scope

Bilateral salpingo-oophorectomy: Remove both fallopian tubes and ovaries. This is the most commonly used surgical scope and can minimize the risk of ovarian and fallopian tube cancer.

Partial salpingo-oophorectomy: In some special cases, such as older patients, poor physical condition or other surgical risk factors, it is possible to consider retaining part of the ovarian tissue. However, this method may leave a certain risk of disease occurrence and needs to be carefully selected.

⑤Postoperative management

Vital signs monitoring: Closely monitor the patient's vital signs after surgery, including body temperature, blood pressure, heart rate, respiration, etc., to ensure the patient's vital signs are stable.

Wound care: Keep the surgical incision clean and dry, change the dressing regularly, and observe the incision for bleeding, exudation, redness and swelling to prevent infection.

Hormone replacement therapy: For premenopausal women, the removal of both ovaries will lead to a sharp decline in estrogen and other hormone levels, and symptoms such as hot flashes, night sweats and osteoporosis may occur. Hormone replacement therapy can be carried out under the guidance of a doctor according to the specific situation of the patient. However, attention should be paid to the possible risks of hormone replacement therapy, such as increasing the risk of breast cancer and cardiovascular diseases.

Follow-up: Regular follow-up after surgery, including gynecological examinations, imaging examinations, tumor marker tests, etc., to detect possible recurrence or metastasis in time. It is generally recommended to conduct the first follow-up 1 - 3 months after surgery, and then follow up every 3 - 6 months.

8.Can fertility-sparing surgery be used in patients with ovarian cancer?

For patients with ovarian cancer, fertility-sparing surgery can be used, but there are strict indications and limitations.

①Indications

Tumor type: It is mainly applicable to early-stage ovarian tumors with low malignant potential, such as borderline ovarian tumors, as well as some early-stage epithelial ovarian cancers, malignant germ cell tumors, etc. For example, for young patients with dysgerminoma who have a need for fertility, fertility-sparing surgery is an important treatment option.

Disease stage: Usually limited to stage I, that is, the tumor is confined to the ovary or fallopian tube, without extra-pelvic metastasis or lymph node metastasis. For example, patients with stage IA ovarian clear cell carcinoma can consider fertility-sparing surgery if other conditions are met.

Patient condition: The patient has a strong desire for fertility, has no other serious comorbidities affecting fertility, and is in good physical condition to tolerate the surgery.

②Surgical methods

For unilateral ovarian tumors, ipsilateral adnexectomy is generally performed, preserving the contralateral ovary and uterus. If both ovaries are involved, it may be necessary to try to preserve some normal ovarian tissue while removing the uterus and bilateral fallopian tubes.

③Precautions

Even after fertility-sparing surgery, close follow-up is still required, including regular gynecological examinations, tumor marker tests, imaging examinations, etc., to detect possible recurrence at an early stage.

For some patients with high-risk recurrence factors, it may be necessary to further supplement surgery or other adjuvant therapies, such as chemotherapy, after completing fertility, depending on the situation.

9.When should secondary cytoreduction be considered in patients with ovarian cancer?

In patients with ovarian cancer, the timing of considering secondary cytoreduction is mainly in the following situations:

①Relapse after initial treatment

For patients with platinum-sensitive recurrent ovarian cancer, if the recurrent lesions can be completely resected and it is expected to achieve no gross residual, secondary cytoreduction can generally be considered. For example, after a period of tumor-free survival after the initial surgery, imaging examinations again suggest resectable recurrent lesions, and tumor markers such as CA125 also increase correspondingly, and the patient's physical condition can tolerate the surgery, then secondary cytoreduction can be considered.

For patients with platinum-resistant recurrence, if, after evaluation, the recurrent lesions are relatively localized and the resection of the recurrent lesions may improve the patient's symptoms and quality of life, secondary cytoreduction can also be carefully considered, but the surgical benefit for such patients may be relatively limited.

②Unsatisfactory cytoreduction in the initial surgery

If the tumor cells could not be cytoreduced to no gross residual or the diameter of the residual lesions was greater than 1 cm due to various reasons in the initial surgery, and after several courses of chemotherapy, the tumor shrinks significantly and imaging examinations show that the remaining tumor is likely to be completely resected, then secondary cytoreduction can be considered at this time to further reduce the tumor burden.

③Progression during maintenance therapy

If the patient has disease progression during maintenance therapy, and the progressive lesions are considered to be completely resectable through evaluation, it can also be a time to consider secondary cytoreduction. However, the overall situation of the patient, including physical function and tumor biological behavior, needs to be comprehensively evaluated.

However, whether to perform secondary cytoreduction or not still needs to be comprehensively evaluated by a multidisciplinary team (including gynecological oncologists, radiologists,

pathologists, etc.) according to the specific situation of the patient, such as age, physical condition, distribution range of the tumor, pathological type, etc.

10.Should ovarian cancer surgery be performed using minimally invasive techniques or laparotomy?

The choice between minimally invasive techniques and laparotomy for ovarian cancer surgery needs to be determined by considering multiple factors:

①Disease factors

Early-stage ovarian cancer: For early-stage (such as stage I) ovarian cancer, if, after strict evaluation, the tumor is confined, there is no obvious pelvic and abdominal metastasis, and the tumor is of moderate size without invasion of surrounding vital organs, minimally invasive surgery is a viable option. Minimally invasive surgery allows for more precise operation, reduces intraoperative bleeding, and lowers the incidence of postoperative complications such as infection, which is beneficial for the patient's postoperative recovery. For example, in the case of early-stage ovarian serous cystadenocarcinoma that meets the above conditions, minimally invasive surgery can be considered.

Advanced-stage ovarian cancer: In advanced-stage ovarian cancer, the tumor usually has a wide range of involvement, and there may be extensive pelvic and abdominal metastases and severe adhesions to surrounding tissues and organs. At this time, laparotomy may be more conducive to the doctor's comprehensive exploration of the abdominal cavity and more thorough cytoreductive surgery to remove all visible tumor lesions as much as possible. For example, in stage III and above ovarian cancer, when the tumor invades multiple organs such as the intestine and bladder, laparotomy can better expose the surgical field and facilitate complex separation and resection operations.

②Patient factors

Physical condition: If the patient is in good physical condition, and the cardiopulmonary function can tolerate the pneumoperitoneum pressure for a long time (minimally invasive surgery requires the establishment of pneumoperitoneum) and there are no serious underlying diseases, both minimally invasive surgery and laparotomy can be considered, but minimally invasive surgery may have a faster recovery. If the patient is older, has poor cardiopulmonary function, or has other serious underlying diseases and has difficulty tolerating pneumoperitoneum or a long

operation, laparotomy may be safer, which can shorten the operation time and reduce the impact of pneumoperitoneum on the respiratory and circulatory systems.

Obesity factor: The pelvic and abdominal anatomical structure of obese patients may be relatively complex, and the operation of minimally invasive surgery is more difficult, which may increase the operation time and the risk of complications. For obese patients with ovarian cancer, laparotomy may be more convenient for the doctor to operate, allowing for a clearer identification of the anatomical structure and reducing surgical damage.

③Medical team factors

Technical level: The experience and technical level of the medical team in minimally invasive surgery are of great importance. If the team is experienced in minimally invasive surgery and can skillfully handle various complex situations, minimally invasive surgery can be preferentially selected for suitable patients with ovarian cancer. Conversely, if the team has limited experience in minimally invasive surgery, for some complex cases, laparotomy may be a more reliable choice to ensure the safety and effectiveness of the surgery.

Equipment conditions: Advanced minimally invasive surgical equipment can provide better support for the operation and help improve the accuracy and safety of the operation. If the hospital has complete and advanced minimally invasive surgical equipment, the feasibility of minimally invasive surgery can be increased. If the equipment is limited, it may restrict the development of minimally invasive surgery.

Medical:

11.Which patients with ovarian cancer should receive neoadjuvant chemotherapy?

The following patients with ovarian cancer should generally consider receiving neoadjuvant chemotherapy:

①Patients with advanced ovarian cancer

Stage Ⅲ and Ⅳ patients: For stage Ⅲ patients with massive ascites, extensive pelvic and abdominal metastases, and the tumor is tightly adhered to surrounding tissues such as the intestine, bladder, and ureter, and it is estimated that it is difficult to completely remove the tumor through the initial surgery. And for stage Ⅳ patients who have distant metastases, such as liver parenchymal metastasis and lung metastasis, neoadjuvant chemotherapy can reduce the tumor size, downstage the tumor, improve the resection rate of subsequent surgery, and reduce surgical complications.

②Patients with huge tumors

Large mass diameter: When the diameter of the ovarian cancer mass is greater than 10 cm, especially when the mass occupies the entire pelvis and even extends to the abdominal cavity, the surgical operation space is small, and it is difficult to completely remove the mass directly. Moreover, it may increase the risk of tumor rupture and implantation metastasis. Neoadjuvant chemotherapy can reduce the volume of the tumor and create better conditions for surgery.

③Patients whose physical condition is intolerant to surgery

With serious underlying diseases: If the patient has severe cardiopulmonary insufficiency, liver and kidney dysfunction, hematological diseases, etc., and is unable to tolerate long-term major surgery, or the surgical risk is extremely high, neoadjuvant chemotherapy can control the tumor progression to a certain extent. At the same time, the patient's physical condition can be adjusted and improved during chemotherapy, and then surgery can be performed when the physical condition permits.

④Patients with special pathological types

Types with high malignancy: Pathological types with high malignancy, rapid growth and early metastasis, such as undifferentiated carcinoma and clear cell carcinoma. Even if they are detected in the relatively early stage of the disease, there may be potential micrometastatic foci.

Neoadjuvant chemotherapy helps to kill potential metastatic foci, reduce the risk of postoperative recurrence and improve the patient's survival rate.

12.When should HIPEC be used in ovarian cancer?

In the treatment of ovarian cancer, hyperthermic intraperitoneal chemotherapy (HIPEC) can be considered in the following situations:

①During primary surgery

Advanced ovarian cancer with massive ascites: If the patient has advanced ovarian cancer and a large amount of ascites is found during surgical exploration, and there may be cancer cells in the ascites. HIPEC can be used to irrigate and perform chemotherapy on the abdominal cavity immediately after tumor resection, which helps to kill residual cancer cells and reduce the risk of postoperative recurrence.

Residual lesions after cytoreductive surgery: During cytoreductive surgery, if there are still visible residual lesions with a diameter less than 1 cm after the operation, or there are microscopic residual cancer cells, HIPEC can use the synergistic effect of hyperthermic effect and chemotherapeutic drugs to better kill residual cancer cells and improve the treatment effect.

②At recurrence

Platinum-sensitive recurrence: For patients with platinum-sensitive recurrent ovarian cancer, that is, recurrence occurs more than 6 months after the end of the last platinum-containing chemotherapy. After resecting the recurrent tumor, HIPEC can be used as an adjuvant treatment to further remove possible residual cancer cells and prolong the patient's progression-free survival.

Solitary recurrent lesion: If the recurrence presents as a solitary lesion in the abdominal cavity, after complete resection of the recurrent lesion, HIPEC can perform chemotherapy on the entire abdominal cavity to reduce the probability of local recurrence again and improve the patient's quality of life and survival rate.

③Special pathological types

Mucinous carcinoma, etc.: For some ovarian cancers with special pathological types, such as mucinous carcinoma, which is prone to intra-abdominal implantation and metastasis, HIPEC can be used before and after surgery, which helps to reduce intra-abdominal tumor implantation and dissemination and control disease progression.

13.Which patients with ovarian cancer should receive bevacizumab for maintenance therapy?

The following patients with ovarian cancer should consider receiving bevacizumab for maintenance therapy:

①Patients with advanced ovarian cancer

Stage Ⅲ and Ⅳ: For patients with stage Ⅲ and Ⅳ ovarian cancer, after achieving complete or partial remission following initial cytoreductive surgery and platinum-based chemotherapy, bevacizumab maintenance therapy can significantly prolong progression-free survival. Because advanced patients have a large tumor burden and a high risk of recurrence. Bevacizumab can delay tumor recurrence by inhibiting angiogenesis and blocking the tumor's nutrient supply.

②Patients with platinum-sensitive recurrence

Respond to platinum-based chemotherapy: If patients with ovarian cancer relapse after the initial treatment and relapse more than 6 months after the end of the last platinum-based chemotherapy (platinum-sensitive recurrence), and achieve remission after re-treatment with platinum-based chemotherapy combined with bevacizumab, then subsequent use of bevacizumab for maintenance therapy can further consolidate the efficacy and reduce the risk of recurrence again.

③Patients with high-risk factors

Residual lesions after cytoreductive surgery: If there are residual lesions after cytoreductive surgery, even if remission is achieved after chemotherapy, the remaining cancer cells may still continue to grow and metastasize. Bevacizumab maintenance therapy helps to inhibit the angiogenesis of residual cancer cells and reduce their chances of proliferation and metastasis.

Slow decline of CA125 or not decreased to normal: During chemotherapy, if the tumor marker CA125 declines slowly, or does not decrease to the normal range at the end of chemotherapy, it indicates that there may still be a large number of cancer cells in the body. Such patients have a high risk of recurrence, and bevacizumab maintenance therapy may bring greater benefits.

Presence of ascites: For patients with ovarian cancer who have ascites, it indicates that the tumor may have invaded the peritoneum and other parts. There may be cancer cells in the ascites,

the condition is relatively complex, and the risk of recurrence is high. Bevacizumab can reduce the production of ascites and inhibit tumor angiogenesis, which is of great significance for the maintenance therapy of such patients.

14.Should patients with recurrent epithelial ovarian cancer who had received one prior PARP inhibitor as maintenance therapy be retreated with a PARP inhibitor?

For patients with recurrent epithelial ovarian cancer who have previously received a poly ADP-ribosyl polymerase (PARP) inhibitor as maintenance therapy, the decision to re-administer a PARP inhibitor requires a comprehensive consideration of multiple factors:

① Tumor-Related Factors

Time of Recurrence: If a patient has a relatively long progression-free survival (PFS) after completing the initial PARP inhibitor maintenance therapy, for example, more than 6 months or even longer, reusing a PARP inhibitor may still have a good therapeutic effect and can be considered. If the time to recurrence is short after the end of the previous treatment, it suggests that the tumor may have developed resistance to PARP inhibitors, and the benefits of reusing it may be limited.

Tumor Biological Characteristics: If the tumor has homologous recombination repair (HRR) gene defects, such as BRCA1/2 gene mutations, reusing a PARP inhibitor may be more likely to be beneficial, as these tumors are more sensitive to the mechanism of action of PARP inhibitors.

② Patient-Related Factors

Physical Condition: The patient's general physical condition and organ function are crucial for tolerating the re-administration of a PARP inhibitor. If the patient is in good physical condition, with normal liver and kidney function, bone marrow function, etc., and can better tolerate the adverse reactions of the drug, reusing it can be considered. If the patient is physically weak, has serious underlying diseases, or experienced severe adverse reactions during the last use of a PARP inhibitor, reusing it may bring greater risks and needs to be considered carefully.

Previous Treatment Response: Review the patient's response to the previous treatment with a PARP inhibitor. If the efficacy was significant during the last use and the adverse reactions were tolerable, reusing it may be a reasonable choice. If severe adverse reactions that were difficult to control occurred during the last use, or if the efficacy was poor, a more cautious assessment is needed before considering reusing it.

③ Diversity of Treatment Options

Availability of Other Effective Therapies: If there are other effective treatment options available, such as new chemotherapeutic drugs, immunotherapeutic drugs, or targeted therapeutic drugs, and these options may have better efficacy and safety in the patient's specific situation, other therapies may be prioritized over reusing a PARP inhibitor. If there are limited alternative treatment options, and reusing a PARP inhibitor has a theoretical basis and potential benefits, it can be chosen after a full assessment.

In general, there is no absolute standard for whether to reuse a PARP inhibitor in such patients. It requires oncology specialists to make a decision based on an individualized comprehensive assessment of the patient's specific situation, including tumor characteristics, physical condition, previous treatment response, and available treatment options, weighing the pros and cons.

15.Should patients with ovarian cancer be treated with immunotherapy or not?

Whether patients with ovarian cancer should be treated with immunotherapy needs to be determined by considering multiple factors:

①Advanced ovarian cancer

No effective alternative: For patients with advanced ovarian cancer, if the disease still progresses after traditional treatments such as surgery and chemotherapy, and there are no other more effective alternative treatments, immunotherapy can be tried. For example, some patients who are unable to undergo secondary surgery and are resistant to chemotherapy drugs such as platinum, immunotherapy may bring certain survival benefits.

Positive for specific biomarkers: If the patient's tumor tissue is detected with specific biomarkers such as high PD - L1 expression, high microsatellite instability (MSI - H) or mismatch repair deficiency (dMMR), immunotherapy may have a good curative effect and can be considered for use.

②Early-stage ovarian cancer

High risk of recurrence: For some patients with early-stage ovarian cancer but at high risk of recurrence, such as those with high-risk factors such as lymphovascular space invasion and poor differentiation although the tumor stage is early, immunotherapy can be explored for maintenance therapy after completing standard surgery and adjuvant chemotherapy to reduce the risk of recurrence, but the evidence in this regard is still being accumulated.

③Patient's physical condition

Good physical condition: The patient's physical condition needs to be able to tolerate the adverse reactions that immunotherapy may bring, such as immune-related inflammatory reactions. Generally, it is required that the patient's cardiopulmonary function and liver and kidney functions are basically normal, and the performance status score is good (such as ECOG score 0 - 1) to better receive immunotherapy.

No severe autoimmune diseases: If the patient has severe autoimmune diseases, such as systemic lupus erythematosus and rheumatoid arthritis in the active stage, the use of immunotherapy may aggravate the condition of autoimmune diseases and is generally not

recommended; if the autoimmune disease is in a stable period, it can be carefully considered after multidisciplinary evaluation.

Surveillance:

16.How should patients with ovarian cancer be followed after treatment is complete?

Follow-up of patients with ovarian cancer after the completion of treatment is of great significance and mainly includes the following aspects:

①Clinical examination

Physical examination: Conduct a comprehensive physical examination regularly, including checking for abdominal masses and ascites, and performing gynecological examinations to evaluate the pelvic condition, etc. Generally, it should be carried out every 3 to 6 months.

Imaging examination: Commonly used ones include ultrasound, CT, and MRI. Ultrasound can be used to check for recurrence in the pelvis and is generally performed every 3 to 6 months. CT or MRI can observe the abdomen, pelvis, and other possible metastatic sites more clearly and is usually performed every 6 to 12 months. If necessary, a PET-CT examination may also be required to check for distant metastases.

②Tumor marker monitoring

CA125: It is one of the most important tumor markers for ovarian cancer. It should be monitored regularly after treatment, generally once every 1 to 3 months. If the CA125 level continues to rise, it may indicate tumor recurrence.

Other markers: Such as HE4, CA153, etc., can also be used as auxiliary monitoring indicators. Analyzing them in combination with CA125 is helpful for a more accurate judgment of the condition.

③Symptom and quality of life assessment

Symptom monitoring: Patients should pay close attention to whether there are new symptoms, such as abdominal pain, abdominal distension, vaginal bleeding, fatigue, etc., and inform the doctor in time if there are any abnormalities.

Quality of life assessment: Doctors should pay attention to the patient's quality of life, including physical function, psychological state, social support, etc. Provide corresponding support and intervention measures when necessary, such as psychological counseling and rehabilitation guidance.

④Genetic testing follow-up

Germline BRCA testing: For patients with a family history or suspected BRCA gene mutations, germline BRCA testing can be performed after treatment to assess the genetic risk and guide the prevention and screening of family members.

Other genetic testing: If genetic testing was carried out to guide targeted therapy, etc. during the treatment process, relevant genetic testing follow-up can also be carried out as needed after treatment to observe changes in the genetic status.

17.How to define platinum-resistant recurrent ovarian cancer?

The definition of platinum-resistant recurrent ovarian cancer is as follows:

①Time criterion: Disease recurrence or progression within 6 months after the completion of platinum-based chemotherapy can be defined as platinum-resistant recurrent ovarian cancer. This is based on the finding in clinical research and practice that tumors that recur within this time frame tend to have a poor response to re-treatment with platinum drugs.

②Treatment response criterion: If the disease continues to progress during the treatment with platinum drugs, including the continuous increase of tumor markers such as CA125, imaging examination showing tumor enlargement or the appearance of new lesions, etc., it is also considered platinum-resistant. Even if the time from the start of treatment to progression may exceed 6 months, this situation also belongs to the category of platinum-resistant recurrent ovarian cancer.

③Special cases: If the patient relapses more than 6 months after the completion of platinum chemotherapy, but the treatment with platinum drugs is ineffective, that is, the tumor continues to progress after the re-use of platinum drugs, this situation can also be classified as platinum-resistant recurrent ovarian cancer, but it is relatively rare, and multiple factors need to be comprehensively judged, such as whether there are other factors affecting the treatment effect.

18.What is the management strategy for biochemical relapse of ovarian cancer?

The management strategies for biochemical relapse of ovarian cancer are as follows:

①Monitoring and assessment

Closely monitor tumor markers: Mainly focus on CA125 and conduct regular (generally every 1 - 3 months) tests to observe its changing trend. Meanwhile, other markers such as HE4 can be combined for a comprehensive evaluation of the condition.

Imaging examination: According to the specific situation, perform ultrasound, CT or MRI and other examinations every 3 - 6 months to check for radiologically visible recurrent lesions. If the tumor marker continues to rise and recurrence is highly suspected, more frequent examinations or further PET - CT examinations may be required.

Comprehensive physical examination: Including gynecological examination, abdominal examination, etc., to evaluate the overall physical condition and pay attention to any suspicious symptoms or signs.

②Treatment decisions

Watch and wait: For low-risk patients with a small increase in tumor markers, no obvious symptoms and no clear lesions found in imaging examinations, the watch and wait strategy can be chosen. Closely monitor the above indicators regularly and observe the changes of the condition.

Initiate treatment：

Ⅰ Systemic treatment: For patients with a rapid increase in tumor markers, those with high-risk factors (such as a short tumor-free survival period after the initial treatment, platinum resistance, etc.) or those with lesions already found by imaging, systemic treatment can be considered. Commonly used regimens include chemotherapy, such as choosing non-platinum single-agent or combination chemotherapy. For patients with specific targets such as BRCA gene mutations, PARP inhibitors can be used for targeted therapy. The combination of anti-angiogenic drugs can also be considered.

Ⅱ Local treatment: If imaging reveals isolated recurrent lesions and the patient's physical condition permits, local treatment methods such as surgical resection or local radiotherapy can be considered to control local lesions and relieve symptoms.

③Supportive treatment

Nutritional support: Ensure that patients receive sufficient nutrition to maintain a good physical condition and tolerate possible treatments. According to the patient's nutritional status, nutritional supplements or enteral and parenteral nutritional support should be provided when necessary.

Psychological support: Help patients cope with the psychological stress and anxiety brought about by recurrence. Psychological counseling, patient mutual assistance and other means can be used to improve patients' psychological resilience and quality of life.

<u>19.What are the independent prognostic factors for ovarian cancer?</u>

The independent prognostic factors for ovarian cancer are as follows:

①Tumor-related factors

Tumor stage: It is one of the most important prognostic factors. In the International Federation of Gynecology and Obstetrics (FIGO) staging, patients with early-stage (stage I and II) ovarian cancer have a relatively good prognosis and a high 5-year survival rate. However, patients with late-stage (stage III and IV) ovarian cancer have a poor prognosis, and the 5-year survival rate is significantly reduced, because late-stage tumors have often spread outside the pelvis or to distant organs.

Histological type: The prognosis of ovarian cancer varies with different histological types. For example, serous ovarian cancer is the most common type. High-grade serous carcinoma is usually more aggressive and has a relatively poor prognosis, while low-grade serous carcinoma and mucinous carcinoma are relatively less aggressive and have a relatively better prognosis.

Tumor grade: High-grade tumor cells are poorly differentiated, grow rapidly, and have strong invasion and metastasis abilities, resulting in a poor prognosis. Low-grade tumor cells are better differentiated, grow relatively slowly, and have a relatively better prognosis.

②Patient-related factors

Age: Generally speaking, younger patients (such as those under 50 years old) have relatively better physical function and tolerance to treatment, and may have a relatively better prognosis. Elderly patients (such as those over 65 years old) often have multiple underlying diseases, poor physical function and immunity, and poor response and tolerance to treatment, resulting in a relatively poor prognosis.

Performance status: Evaluated by the Eastern Cooperative Oncology Group (ECOG) performance status score, etc. The lower the score (such as 0 - 1), the better the patient's physical condition, and they can better tolerate treatments such as surgery and chemotherapy, with a relatively better prognosis. A high score (such as 2 or above) indicates a poor physical condition of the patient and a poor prognosis.

Comorbidities: Patients with underlying diseases such as cardiovascular diseases, diabetes, and chronic pulmonary diseases may affect the implementation and effectiveness of treatment and increase the risk of treatment-related complications, thus leading to a poor prognosis.

③Treatment-related factors

Degree of surgical resection: An ideal cytoreductive surgery can remove as much tumor tissue as possible, with the remaining lesions being as small as possible. If there is no visible residual lesion, the patient's prognosis is significantly better than that of patients with large residual lesions.

Chemotherapy regimen and response: Patients who are sensitive to platinum-based chemotherapy regimens have a relatively good prognosis. If patients have severe adverse reactions during chemotherapy, resulting in the inability to carry out chemotherapy as planned, or if the tumor is resistant to chemotherapy drugs, it will affect the prognosis.

Maintenance treatment: For patients who achieve complete or partial remission after initial treatment, maintenance treatment with PARP inhibitors and others can prolong the progression-free survival and improve the prognosis.

④Molecular biological factors

BRCA gene mutation: Ovarian cancer patients with BRCA1 or BRCA2 gene mutations may be more sensitive to platinum-based chemotherapy drugs and have a better initial treatment effect, but may face more complex treatment situations after recurrence. Overall, their prognosis is different from that of wild-type patients, and they are more likely to benefit from PARP inhibitor treatment.

Gene expression profile: Some specific gene expression characteristics, such as the high expression of certain genes related to cell proliferation, invasion and angiogenesis, often indicate a poor prognosis, while the high expression of some tumor suppressor genes may be related to a better prognosis.

Protein markers: The expression of proteins such as p53 and Ki-67 is also related to prognosis. Abnormal expression of p53 protein or high expression of Ki-67 is usually associated with high proliferative activity of tumors and poor prognosis.

20.How to manage chemotherapy-associated adverse events in patients with ovarian cancer?

The management methods for chemotherapy - associated adverse events in patients with ovarian cancer are as follows:

①Hematological adverse reactions

Myelosuppression: Regularly monitor the blood routine and take different measures according to the degree of reduction of white blood cells, red blood cells, platelets, etc. When the white blood cells are too low, granulocyte colony - stimulating factor can be used to increase the white blood cells. Protective isolation may be necessary to prevent infection. In case of severe anemia, blood transfusion or erythropoietin can be considered. When the platelet count is significantly reduced, pay attention to preventing bleeding and transfuse platelets if necessary.

Bleeding tendency: Observe whether the patient has symptoms such as petechiae, ecchymoses, epistaxis, gingival bleeding, etc. If there is a bleeding tendency, reduce activity, avoid collisions, and use hemostatic drugs if necessary.

②Digestive system adverse reactions

Nausea and vomiting: Anti - emetic drugs such as 5 - hydroxytryptamine receptor antagonists and dopamine receptor antagonists can be used prophylactically before chemotherapy. In terms of diet, it is recommended to have small and frequent meals and choose light and easily digestible foods, avoiding greasy and irritating foods. When severe vomiting leads to dehydration or electrolyte disorders, intravenous fluid replacement is required to maintain water and electrolyte balance.

Diarrhea: Mild diarrhea can be alleviated by adjusting the diet, such as eating low - residue and low - fiber foods, and at the same time, replenish water and electrolytes. In case of severe diarrhea, anti - diarrhea drugs such as montmorillonite powder should be used. If it is infectious diarrhea, antibiotics are also needed for treatment.

Oral mucositis: Keep the oral cavity clean and use mild oral care products, rinse the mouth after meals. When the symptoms are severe, drugs that promote mucosal repair, such as Kangfuxin Liquid, can be used.

③Neurological adverse reactions

Peripheral neuropathy: Manifested as limb numbness, pain, etc. Nerve - nourishing drugs such as mecobalamin can be used. At the same time, pay attention to the patient's safety to prevent accidents such as falls caused by abnormal sensations.

Central nervous system symptoms: In case of headache, dizziness, memory loss, etc., pay attention to rest and ensure sufficient sleep. When the symptoms are severe, symptomatic treatment can be given, such as using painkillers.

④Urinary system adverse reactions

Nephrotoxicity: Ensure sufficient water intake during chemotherapy and carry out hydration and diuresis if necessary to reduce the burden on the kidneys. Regularly monitor renal function and adjust the dosage of chemotherapy drugs according to the renal function.

Hemorrhagic cystitis: Encourage patients to drink more water to increase urine output and flush the urinary tract. When the symptoms are severe, hemostatic and bladder mucosa - protecting drugs can be used.

⑤Other adverse reactions

Allergic reaction: Take preventive measures before chemotherapy, such as using anti - allergic drugs. Closely observe whether the patient has allergic symptoms such as rashes, itching, and dyspnea during chemotherapy. Once an allergic reaction occurs, stop chemotherapy immediately and give anti - allergic treatment, such as using glucocorticoids, epinephrine, etc.

Hair loss: Inform the patient in advance that hair loss is a common adverse reaction of chemotherapy to prepare them psychologically. It is advisable to suggest that patients wear wigs, hats, etc. for modification.
