# Supplementary material for: Decoding AI Competence: Benchmarking Large Language Models (LLMs) in Ovarian Cancer Diagnosis and Treatment—A Systematic Evaluation of Generative AI Accuracy and Completeness
Source: Diagnostics (Basel). 2026 Feb 20;16(4):616. doi: 10.3390/diagnostics16040616 (PMC12939671; doi:10.3390/diagnostics16040616)
Supplement: Supplementary file 1 [file diagnostics-16-00616-s001.zip › FileS6-questions.pdf]

- **Risk Factors and Prevention:**
  - What are the risk factors for ovarian cancer?
  - What genetic and molecular testing is recommended in patients with ovarian cancer?
  - How much do BRCA mutations increase risk for ovarian cancer?
  - Who should be tested for BRCA mutations?
  - How can you prevent cancer if you test positive for BRCA mutations?

- **Surgical:**
  - What are the principles of surgery for newly diagnosed invasive epithelial ovarian cancer involving the pelvis and upper abdomen (stage ≥IIB)?
  - What is the surgical protocol for risk-reducing salpingo-oophorectomy?
  - Can fertility-sparing surgery be used in patients with ovarian cancer?
  - When should secondary cytoreduction be considered in patients with ovarian cancer?
  - Should ovarian cancer surgery be performed using minimally invasive techniques or laparotomy?

- **Medical:**
  - Which patients with ovarian cancer should receive neoadjuvant chemotherapy?
  - When should HIPEC be used in ovarian cancer?
  - Which patients with ovarian cancer should receive bevacizumab for maintenance therapy?
  - Should patients with recurrent epithelial ovarian cancer who had received one prior PARP inhibitor as maintenance therapy be retreated with a PARP inhibitor?
  - Should patients with ovarian cancer be treated with immunotherapy or not?

- **Surveillance:**
    - How should patients with ovarian cancer be followed after treatment is complete?
    - How to define platinum-resistant recurrent ovarian cancer?
    - What is the management strategy for biochemical relapse of ovarian cancer?
    - What are the independent prognostic factors for ovarian cancer?
    - How to manage chemotherapy-associated adverse events in patients with ovarian cancer?
